# A high-performance oxygen evolution catalyst in neutral-pH for sunlight-driven CO_2_ reduction

**DOI:** 10.1038/s41467-019-12009-8

**Published:** 2019-09-09

**Authors:** Li Qin Zhou, Chen Ling, Hui Zhou, Xiang Wang, Joseph Liao, Gunugunuri K. Reddy, Liangzi Deng, Torin C. Peck, Ruigang Zhang, M. Stanley Whittingham, Chongmin Wang, Ching-Wu Chu, Yan Yao, Hongfei Jia

**Affiliations:** 1 Toyota Research Institute of North America, Ann Arbor, MI 48105 USA; 2 Chemistry and Materials, Binghamton University, Binghamton, NY 13902 USA; 3 Department of Mechanical Engineering and Materials Science, University of Pittsburgh, Pittsburgh, PA 15261 USA; 4 Enli Technology Co. Ltd., Kaohsiung City, 82151 Taiwan; 5 Texas Center for Superconductivity and Department of Physics, University of Houston, Houston, TX 77204 USA; 6 Environmental Molecular Sciences Laboratory, Pacific Northwest National Laboratory, Richland, WA 99352 USA; 7 Lawrence Berkeley National Laboratory, Berkeley, CA 94720 USA; 8 Department of Electrical and Computer Engineering and Texas Center for Superconductivity (TcSUH), University of Houston, Houston, TX 77204 USA

**Keywords:** Catalyst synthesis, Solar fuels, Electrocatalysis, Atomistic models

## Abstract

The efficiency of sunlight-driven reduction of carbon dioxide (CO_2_), a process mimicking the photosynthesis in nature that integrates the light harvester and electrolysis cell to convert CO_2_ into valuable chemicals, is greatly limited by the sluggish kinetics of oxygen evolution in pH-neutral conditions. Current non-noble metal oxide catalysts developed to drive oxygen evolution in alkaline solution have poor performance in neutral solutions. Here we report a highly active and stable oxygen evolution catalyst in neutral pH, Brownmillerite Sr_2_GaCoO_5_, with the specific activity about one order of magnitude higher than that of widely used iridium oxide catalyst. Using Sr_2_GaCoO_5_ to catalyze oxygen evolution, the integrated CO_2_ reduction achieves the average solar-to-CO efficiency of 13.9% with no appreciable performance degradation in 19 h of operation. Our results not only set a record for the efficiency in sunlight-driven CO_2_ reduction, but open new opportunities towards the realization of practical CO_2_ reduction systems.

## Abstract (web summary)

Solar-to-fuel conversion under mild conditions offers a renewable means to store energy. Here authors report a Brownmillerite oxide for neutral-pH oxygen evolution that, when integrated with an anodized silver cathode, enables a 13.9% energy-conversion efficiency for light-driven CO_2_ reduction

## Introduction

The increasing atmospheric carbon dioxide (CO_2_) level has alarmed the urgent demand of action to mitigate the substantial consequence on our climate. It promotes extensive interest in the development of green alternatives to fossil fuels. A promising approach to meet this challenge is to store solar energy in chemical bonds through sunlight-driven reduction of CO_2_^1^. This process pairs two half-reactions of CO_2_ reduction (CO_2_R) and oxygen evolution reaction (OER) and powers the electrochemical cell using the photocurrents generated from one or more light absorbers. Significant improvements have been achieved in studying CO_2_R and OER in half-cells^2,3^, and various valuable carbon-based products have been synthesized in the lab demonstration of integrated devices, including carbon monoxide (CO), formate, hydrocarbons, and oxygenates^4–10^.

In the sunlight-driven electrolysis, the overall efficiency to convert solar energy into chemicals are critically determined by the factors of the performance of light absorber, the ohmic and Nernstian losses, and, the most importantly, the activity of catalysts to drive kinetically sluggish reactions^11,12^. For CO_2_R, the dissolution of CO_2_ in high pH solution forms less electrochemically active bicarbonate or carbonate and the reduction in low pH faces the competition with hydrogen evolution, hence restricting the most effective environment in neutral pH. On the other hand, for oxygen evolution non-noble metal oxide catalysts work most efficiently under strongly alkaline conditions and experience detrimental cation leaching that severely depresses the activity in neutral solution^13^. Furthermore, the accumulation of the leached metal species onto the cathode can be poisoning to CO_2_R catalyst; hence the cation leaching, even in trace amount, significantly affects the long-term stability of the integrated system^7^. Thus, searching an oxygen evolution catalyst with simultaneously high catalytic activity and compositional stability for integrated CO_2_ reduction in neutral pH remains a critical open challenge to improve the overall solar to fuel (STF) efficiency. Previous studies widely used noble metal oxide such as IrO_2_ to catalyze OER in neutral pH;^4–6,14^ but the moderate activity of IrO_2_ limited the STF efficiency below 7%^11^. Alternately, this challenge was circumvented by isolating CO_2_R and OER in different pH-valued solutions using bipolar membrane (BPM)^8–10^. Whereas the introduction of BPM allowed the operation of CO_2_R and OER in optimal environments to achieve higher STF efficiency over 10% and potentially benefited the separation of product gases, it not only caused additional membrane-derived voltage losses and raised extra complexity of optimization^14–16^, but the ion crossover due to imperfection of the BPM is still a concern for a long-term operation^8^.

Here, we report a significant improvement of STF efficiency enabled by the discovery of a highly active OER catalyst in neutral pH. The OER catalyst reported herein, Sr_2_GaCoO_5_ (SGC), belongs to the family of Brownmillerite oxides, with the general formula of ABO_2_._5_ or A_2_B’BO_5_, where A is a larger sized cation, B and B’ are smaller cations octahedrally and tetrahedrally bonded to oxygen, respectively. As an oxygen-deficient derivative of perovskite, the crystalline structures of Brownmillerite oxides include several variations of oxygen vacancy ordering^17,18^. The compositional degree of freedom, as well as the structural polymorphism of Brownmillerite oxides, offers a great platform to tune the functionality in various oxygen-related applications, such as oxygen storage materials^19^, heterogeneous catalysts^20,21^ and oxygen-ion conductors;^22,23^ but the usage of these compounds as OER catalyst has seldom been explored^24,25^. In the current study, we found that SGC had the specific activity about one order of magnitude higher than that of IrO_2_ and showed superior durability without observable performance degradation in neutral pH. Paring the SGC anode and anodized silver (a-Ag) cathode and coupling the electrolysis cell with triple-junction GaInP/GaInAs/Ge solar cell in a BPM-free device, the integrated device achieved the average solar-to-CO efficiency of 13.9%, setting a record of STF for sunlight-driven CO_2_ reduction.

## Results

### Synthesis and structure of SGC

**Fig. 1:**
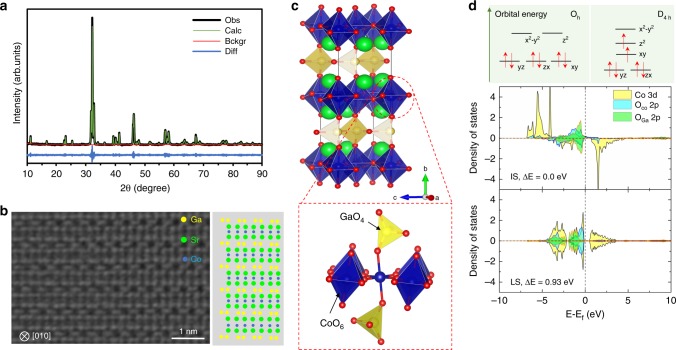
Crystalline and electronic structure of Sr_2_GaCoO_5_. **a** X-ray diffraction pattern and the Rietveld refinement results. The discrepancy factors are R_p_ = 7.3% and R_wp_ = 9.73%. **b** HAADF*-*STEM image of Sr_2_GaCoO_5_. The right-side model illustrates the observed ordering of cations that matches the Brownmillerite structure. **c** Graphic depiction of the crystal structure and bonding environment around the cobalt cation. Cobalt, gallium, strontium, and oxygen are colored as blue, yellow, green and red, respectively. **d** Splitting of Co^3+^ d-orbitals in O_h_ and D_4h_ crystal field and the calculated density of states projected on Co d states, p states of oxygen that bridges two CoO_6_ (O_Co_) and that bridges CoO_6_ and GaO_4_ (O_Ga_). The intermediate spin configuration is 0.93 eV per formula unit more stable than the low spin configuration

The synthesis of SGC was carried out in a solid-state reaction route (see the Methods for more details). The Rietveld refinement of X-ray diffraction (XRD) pattern showed a pure orthorhombic Brownmillerite structure of space group Icmm for the as-synthesized product (Fig. 1a and see Supplementary Table 1 for the refined lattice parameters) with no XRD-detectable impurity. In the structure model, oxygen atom is missed from octahedral GaO_6_ in a normal perovskite, resulting in CoO_6_ octahedra and GaO_4_ tetrahedra stacked alternatingly, which are clearly visible in the high-angle annular dark-field scanning transmission electron microscopy (HAADF-STEM) image (Fig. 1b). Interestingly, the Rietveld refinement suggested nearly full occupancy of Co and Ga at B and B’ site, respectively, in contrast to the normally observed partial occupancies at both sites in other Brownmillerite oxides^17,18^. The full occupancy of Co at octahedral site reduced the distribution of cobalt in less active tetrahedral site^26^, hence improving the utilization of active species.

### OER performance

In a normal perovskite such as LaCoO_3_, cobalt is bound to six CoO_6_ units in a crystal field with an O_h_-like symmetry, favoring a diamagnetic ground state of low spin (LS) Co^3+^ with six electrons in the t_2g_ orbitals and empty e_g_ states^27,28^. In the Brownmillerite SGC two of six surrounding CoO_6_ units are replaced by GaO_4_, lowering the O_h_ symmetry to D_4h_ (Fig. 1c). As a result, the degeneracy of both the t_2g_ and e_g_ orbitals is broken (Fig. 1d), stabilizing the ground state of Co^3+^ in intermediate spin (IS, t_2g_^5^e_g_^1^) configuration^29^, as shown by the electronic structure calculations (Fig. 1d and Supplementary Fig. 1) and confirmed by the magnetization measurements (Supplementary Fig. 2). The stabilization of IS-Co^3+^ crucially benefits the OER activity as the occupancy of e_g_ filling in Co^3+^ increases from theoretically zero in LS state to approaching the optimal value of ~1.2 for OER catalyst^30–32^. Furthermore, the Jahn-Teller effect caused by IS-Co^3+^ elongates Co–O bonds along [010] direction (2.26 Å versus 1.97 Å for Co–O bonds along (010) plane), lifting the corresponding oxygen p-state towards Fermi level (green area in Fig. 1d). Both these effects are positive to enhance the OER performance^32–34^. Using the computational hydrogen electrode method^35^, the activities of two cobalt-containing Brownmillerite oxides, SGC and Sr_2_AlCoO_5_ (SAC), were predicted in the proximity of the optimum of OER catalysts (Supplementary Figs. 3 and 4). In fully agreement with this theoretical picture, SGC and SAC showed remarkably high OER activities in alkaline solution (0.1 M KOH, pH 13, Supplementary Fig. 5). In particular, the overpotential (η) of SGC at the current density of 500 μA·cm^−2^_oxide_ (normalized to the oxide surface areas from Brunauer–Emmett-Teller measurements, BET) was 0.33 V measured using a glassy carbon rotating disk electrode (GC-RDE), which was lower than several perovskite catalysts (0.35–0.41 V)^36^, and comparable to double perovskite (0.29–0.33 V) and recently reported CaCu_3_Fe_4_O_12_ catalyst (0.31 V, see Supplementary Figs. 6 and 7 for details)^32–34^.

**Fig. 2:**
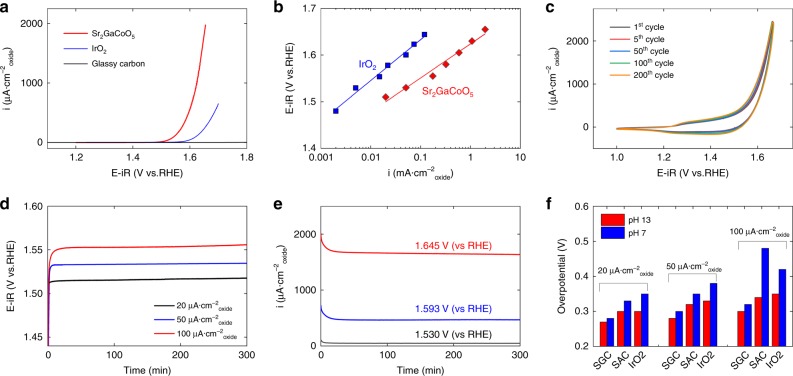
Performance of Sr_2_GaCoO_5_ towards oxygen evolution in neutral-pH. **a** Linear sweep voltammogram of Sr_2_GaCoO_5_, commercial IrO_2_ and blank electrode with only glassy carbon and ink. All current densities were normalized based on the surface area measured in Brunauer–Emmett–Teller analysis, 8.4 m^2^·g^−1^ for Sr_2_GaCoO_5_ and 32.0 m^2^ g^−1^ for IrO_2_. The current for the blank electrode was normalized to the surface area of Sr_2_GaCoO_5_ loaded on the electrode. **b** Tafel plot of Sr_2_GaCoO_5_ and commercial IrO_2_. The data is fitted with the equation $i = i_0{\mathrm{exp}}[\alpha \left( {V - V_0} \right)]$, where $i_0$ is the preexponential factor and *V*_*0*_ is the equilibrium voltage. **c** Cyclic voltammetry of Sr_2_GaCoO_5_. No appreciable change was observed during 200 repeated scans. **d** Galvanostatic experiments at constant current densities. The overpotentials at constant current densities of 20, 50, and 100 μA·cm^−2^_oxide_ was stabilized at 0.28, 0.30 and 0.32 V, respectively. **e** Potentiostatic experiments at constant voltages. The capacity retention after five hours of operation was 94–96%. **f** Comparison of the overpotentials for Sr_2_GaCoO_5_, Sr_2_AlCoO_5_, and IrO_2_ at the same current densities in pH 7 and pH 13 solutions

The high OER activity of SGC persisted when the measurement was performed in pH-neutral solution, a mixture of 0.4 M NaH_2_PO_4_ and 0.6 M Na_2_SO_4_ with pH tuned to 7.0 by proper amount of NaOH. Due to the limited literature data on the OER activity in neutral solutions, we also measured the performance of the golden standard catalyst, IrO_2,_ and thoroughly compared it to that of SGC. In the linear sweep voltammogram, SGC displayed an earlier onset potential and exhibited higher current density than commercial IrO_2_ toward oxygen evolution reaction (Fig. 2a). At the current density of 50 μA·cm^−2^_oxide_, the SGC required an η of 0.30 V, which was 0.07 V and about 0.18 V lower than that of IrO_2_ and a recently discovered NiCoFeP oxyhydroxide catalyst^37^, respectively. The iR-corrected Tafel plot of SGC and IrO_2_ further confirmed the higher intrinsic OER activity of SGC throughout the measured potential range (Fig. 2b). The fitted Tafel slope was 75 mV·decade^−1^ for SGC and 87 mV·decade^−1^ for IrO_2_, while the exchange current density of SGC was 5.5 × 10^–6^ mA·cm^−2^_oxide_, about two times higher than that of IrO_2_ (2.5 × 10^−6^ mA·cm^−2^_oxide_). Using these results, the intrinsic activity of SGC is about 7.8–18.3 times to that of IrO_2_ at the applied potential of 1.53–1.73 V vs RHE, and the mass activity is 2.0–4.8 times to that of IrO_2_. In Supplementary Fig. 8, the OER performance recorded in 0.1 M KHCO_3_ electrolyte showed only marginal difference with that in NaH_2_PO_4_/Na_2_SO_4_ solution, further demonstrating the intrinsically high OER activity of SGC in neutral conditions instead of being sensitive to the choice of electrolyte.

The OER performance of SGC was highly sustainable for a long-time operation, as shown in the cyclic voltammetry (CV), galvanostatic and potentiostatic tests (Fig. 2d–f). The current density at 1.65 V vs RHE was measured at 1.7 mA/cm^2^_oxide_ with the retention after five hours of operation at 96%, demonstrating the superior stability to deliver high current at moderate overpotentials.

The comparison of pH 7 and pH 13 results showed that the OER performance of SGC barely depends on the pH value of electrolyte. At current densities no higher than 100 μA·cm^−2^_oxide_ the overpotential in pH 7 solution was only 0.01–0.02 V higher than that in pH 13 (Fig. 2f). For comparison, the η of IrO_2_ in pH 7 was 0.05–0.07 V higher than those in pH 13 in the same current density range. Even at the current density of 500 μA·cm^−2^_oxide_ (10.5 mA·cm^−2^_geo_) the measured η of SGC was only 0.04 V higher in pH 7 solution. This weak pH-dependence of η was seldom observed in other perovskite catalysts. For example, for perovskite Ba_0_._5_Sr_0_._5_Co_0_._8_Fe_0_._2_O_3-δ_, LaMO_3_ (M=Mn, Fe, Co, and Ni) and double perovskite PrBaCo_2_O_5+δ_, the OER activities are severely reduced due to the detrimental dissolution of cations in neutral pH even at the current density as low as 5 μA·cm^−2^_oxide_^13^, resulting in the increase of η by over 0.1–0.3 V compared to that in alkali conditions^13,38^. As Tsuji et al. reported, the OER activity of Brownmillerite-type Ca_2_FeCoO_5_ was strongly affected by the concentration of KOH solution^25^. Another Brownmillerite oxide studied in the current work, SAC, had the overpotential increased by ~0.03 V in neutral pH at low current densities, but exhibited a large increase of η at 100 μA·cm^−2^_oxide_ (Fig. 2f and Supplementary Figs. 9–11), which signaled severe performance degradation at moderate to high current densities. Therefore, the observed weak pH-dependence of η should be related to both the Brownmillerite structure and the unique composition of SGC.

While the GC-RDE setup allowed us to firmly establish the intrinsic activity and stability of SGC for oxygen evolution in neutral solution, we also evaluated the potential of using SGC towards practical applications. To do this we drop-casted the catalyst ink on a carbon paper electrode and measured the performance in a three-electrode setup. For the catalyst loading of 1 mg·cm^−2^_geo_, the measured overpotential was 0.38 V at the current density of 10 mA·cm^−2^_geo_ and barely changed in the testing of 72 h (Supplementary Fig. 12). Due to the mass transfer resistance in the porous electrode, this value was about 0.05 V higher than that estimated from the GC-RDE measurements. Increasing the loading effectively decreased the overpotential at the same current density. For the loading of 3 mg·cm^−2^_geo_^37^, the overpotential was reduced to 0.35 V, only 0.02 V higher than the NiCoFeP oxyhydroxide catalyst. Even at a high current density of 100 mA·cm^−2^_geo_, the voltages remained highly stable with an increase of less than 0.015 V in the testing of 32 h. While engineering the loading and electrode structure to optimize the performance will be left to future work, these results clearly demonstrated the remarkable performance of SGC in conditions for practical applications.

### Mechanistic insights

**Fig. 3:**
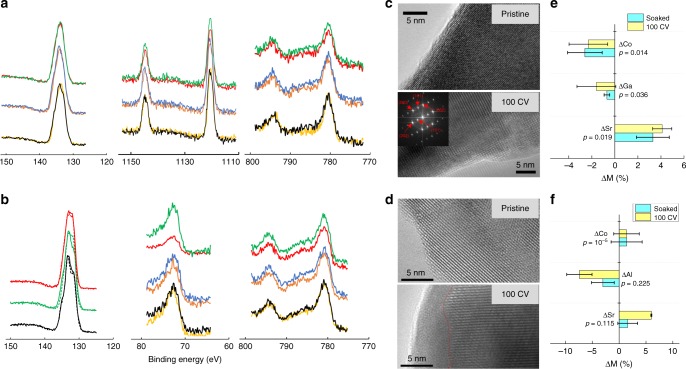
Structural and compositional stability of Sr_2_GaCoO_5_ under oxygen evolution conditions in neutral-pH. **a** X-ray photoelectron spectroscopy results for Sr 3d (left), Ga 2p (middle) and Co 2p (right) signal in Sr_2_GaCoO_5_. **b** X-ray photoelectron spectroscopy results for Sr 3d (left), Al 2p (middle) and Co 2p (right) signal in. Each figure contains three groups of spectra, corresponding to pristine sample (bottom), sample soaked in the electrolyte for four hours (middle) and sample after 100 cyclic voltammetry cycles from 1.0 to 1.7 V (top). For each group, the bottom one shows the signal from fresh surface and the top one shows the signal after Ar^+^ sputtering for 16 min The signal of Al is magnified by 10 times for better visualization. **c**, **d** HRTEM images of Sr_2_GaCoO_5_ (**c**) and Sr_2_AlCoO_5_ (**d**) for pristine sample and for sample after 100 cyclic voltammetry scans from 1.0 to 1.7 V. The insert in **c** shows the indexed selected area electron diffraction. The red line marked the boundary between the crystalline and amorphous regions. **e** Difference of cation content between on the surface and in the bulk of Sr_2_GaCoO_5_ from 10 energy dispersive spectroscopy measurements. **f** Difference of cation content between on the surface and in the bulk of Sr_2_AlCoO_5_ from 15 energy dispersive spectroscopy measurements. The statistical significance of the difference between soaked and cycled samples was evaluated in the equivalence test, designed as two one-side *t*-tests to test if the difference is larger than θ or smaller than −θ, where the acceptance criterion θ was chosen to be the average standard deviation of metal content, 2%, for Sr_2_GaCoO_5_. Due to the obviously larger change of Sr and Al contents in Sr_2_AlCoO_5_ samples, we used a larger acceptance criterion θ, 5%, in the test. The largest *p*-value at 95% confidence level is reported on the graph

These results highlighted an intrinsically high, sustainable and weakly pH-dependent performance of SGC to catalyze oxygen evolution. To shed light on these observations, we conducted the X-ray photoelectron spectroscopy (XPS) studies to probe the effect of OER operation on the chemical states of cations in SGC. Whereas the XPS spectra clearly showed the surface adsorption of hydroxyl groups after soaking the sample in the electrolyte for four hours or after 100 CV cycles (Supplementary Fig. 13), little difference was observed for Sr, Co, and Ga signals after contacting the aqueous electrolyte see Fig. 3a. More importantly, the XPS spectra collected on the fresh surface and after Ar^+^ etching for ~10 nanometers (16 min, assuming an etching rate of around 0.5−1 nm/min^39^) did not exhibit any appreciable difference on metal signals, indicating no evidence for surface reconstruction beyond XPS-detectable level that tuned the chemical state of active species for OER activity as observed in other Co-based OER catalysts^40–44^. By integrating the area of the Co 2p and Ga 2p peak, the quantitative difference between the cation contents on the surface and in the bulk was estimated to be less than 5% (Supplementary Fig. 14), which was likely within the instrumental accuracy of estimating elemental content with XPS spectra. Therefore, the XPS results suggested that SGC must be chemically stable against possible OER-induced structural and compositional changes in neutral solutions. On the contrary, the XPS spectra of SAC showed the decrease of Al signals on the surface of CV cycled sample (Fig. 3b), suggesting the appreciable dissolution of Al during the OER operation.

To further study the influence of OER operation on the surface and bulk structure of SGC, we investigated the crystalline structures using high-resolution transmission electron microscopy (HRTEM). Well crystallized surface was observed for as-synthesized samples. After 100 CV cycles in neutral pH, the particle remained high crystallinity with lattice fringes extending to the surface (Fig. 3c and additional images in Supplementary Fig. 15). Based on the energy dispersive spectroscopy (EDS), we found that the OER operation did not introduce any appreciable difference on the surface composition than simply soaking SGC in the electrolyte (Fig. 3e and Supplementary Tables 2–4), in consistent with the XPS results. These results confirmed the high structural and compositional stability of SGC during OER operation in neutral environment. On the contrary, apparent amorphous layer coated on the crystalline SAC particles was observed after CV cycling in neutral pH (Fig. 3d). In consistent with the XPS results shown in Fig. 3c, d, the EDS showed a statistically significant decrease of Al amount in the surface layer (Fig. 3f and Supplementary Tables 5–7), therefore attributing the amorphourization to Al-dissolution induced destruction of crystalline surface.

Combining the XPS, HRTEM, and EDS results provided the mechanistic picture of the high OER performance of SGC in neutral solution. Due to the presence of IS-Co^3+^ ions in their Brownmillerite structures, both SGC and SAC have intrinsically high activity towards oxygen evolution in alkaline solutions. However, in neutral solutions, only SGC maintains the active crystalline surface for oxygen evolution while the surface of SAC experiences Al-leaching induced amorphourization. Consequently, the activity of SAC showed appreciable degradation in neutral solution especially at high current conditions, while the performance of SGC is highly sustainable and nearly pH-independent. Considering the compositional and structural similarity between SGC and SAC, the difference in stability is attributed to the thermodynamic driving force of dissolving aluminum oxide and gallium oxide at the OER operation condition in neutral pH, as indicated by the wider range of stability for solid gallium oxide in the computational Pourbaix diagrams (Supplementary Figs. 16–18). We note that this simple argument does not consider the complexity brought by the co-existence of Sr and Co or the kinetics of cation leaching. A detailed mechanistic picture for the stability of Brownmillerite oxides in neutral-pH OER should be the key subject of future studies.

### Sunlight-driven CO_2_ reduction

**Fig. 4:**
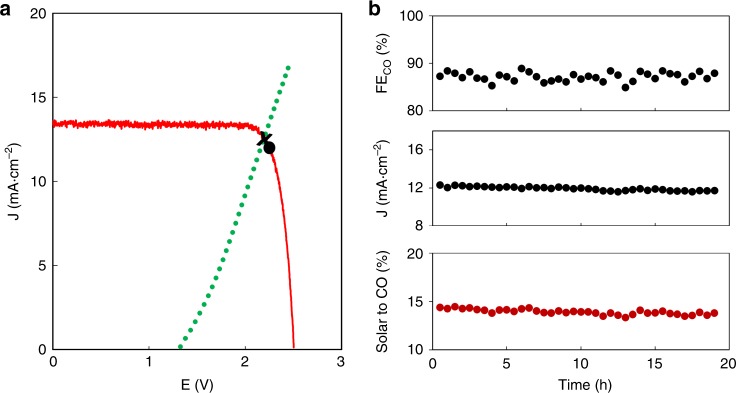
Sunlight-driven CO_2_ reduction using Sr_2_GaCoO_5_ anode for oxygen evolution and anodized silver cathode for CO_2_ reduction. **a** Current density–voltage characteristics of the photovoltaic (red) and electrolysis cell (green). The photovoltaic performance is shown under illumination of standard AM 1.5G solar light and 100 mW·cm^−2^ intensity (1 sun) and its maximum power point is marked by a black dot. The calculated intersection is shown as the black cross. **b** Faradaic efficiency, solar current density and STF_CO_ efficiency for the total operation of 19 h. The fluctuation of STF_CO_ was mainly attributed to the change of Faradaic efficiency by as much as 4% due to the sensitive CO selectivity on the potential applied on a-Ag cathode

We then proceeded the evaluation by pairing the SGC anode for OER and a-Ag cathode for CO_2_R in an BPM-free electrolysis cell, as illustrated in Supplementary Fig. 19. We used a-Ag for its high selectivity towards CO generation under a moderate overpotential around 0.5 V^45^. Taken into account the overpotentials for both electrodes, an operating voltage around 2.2 V is expected., A triple junction of GaInP/GaInAs/Ge photovoltaic with an open-circuit voltage of 2.51 V was therefore used to effectively drive the electrolysis. The room temperature current density–voltage (J–V) profile of the triple junction intersected with the J–V curves of the electrochemical system close to the maximum power point of solar-to-electric energy conversion (Fig. 4a), allowing effective conversion of solar energy into electricity. For comparison, using the same solar cell and a-Ag cathode but commercial IrO_2_ anode for OER, the J–V curves intersected farther from the maximum power point at lower current densities, leading to poorer utilization of solar energy for conversion (Supplementary Fig. 20).

The high compositional stability of SGC in neutral solution reduced the possible metal ion contamination on CO_2_-reduction cathode, allowing the sustainable operation without necessarily refreshing the electrolyte for a long period. We, therefore, carried out the electrolysis over the total course of 19 h, the longest time of operation in the sunlight-driven CO_2_ reduction and far exceeding those employing non-noble metal catalysts for OER in neutral-pH solutions^7,37^. The integrated system delivered a nearly constant current density of 11.9 mA·cm^−2^ at an average voltage of 2.26 V (Fig. 4b). The current and voltage fluctuated less than 6% and 3%, respectively, not only confirming the stable performance of the BPM-free device but also reflecting the superior stability of both electrodes under real operating conditions. By monitoring the electrode potential using reference Ag/AgCl electrode, the average iR-uncorrected overpotential was estimated to be 0.56 and 0.37 V at the a-Ag cathode and SGC anode, respectively. Considering the extra mass transfer resistance and the Ohmic loss in the integrated device, both values were in good agreement with those estimated from the half-cell measurements (Supplementary Fig. 21), indicating little performance degradation from the integration of half-cells. For comparison, devices using BPM to isolate CO_2_R and OER in different pH-valued solutions had extra voltage losses in the order of 0.2–0.5 V due to the resistance of BPM and the necessary overpotential for water dissociation^8,10^.

The Faraday efficiency for CO generation varied between 85% and 89% and the average value in the 19-h period of operation was 87.2%. The solar-to-CO conversion (STF_CO_) efficiency was peaked at 14.4% and the average over 19 h was 13.9%. Taking into account both products of CO and H_2_, the STF efficiency peaked at 16.3% and the average was 15.6%. For comparison, using IrO_2_ anode for OER, the STF_CO_ efficiency was 9.7% and the STF efficiency was 10.9% after 5 h of operation, about 30% less than the same device using SGC anode. Using IrO_2_ anode, oxidized gold electrode cathode and perovskite solar cell, Schreier et al. achieved solar to CO efficiency of 6.5%^4^. Therefore, we may attribute the usage of triple junction solar cell and a-Ag cathode in current study contributed to ~3% of efficiency improvement, while using SGC for OER further improved the efficiency by ~4%. Table 1 summarizes the current results in comparison with previous studies of sunlight-driven CO_2_ reduction devices. Compared to other experimental results, the STF was improved by at least 130% compared to IrO_2_-involved systems, and 12% for BMP-based devices, hence setting the benchmark record for future improvements.

**Table 1:** Comparison of the performance of sunlight-driven CO_2_ reduction devices

Device configuration					Efficiency		Duration	Ref.
OER	CO_2_RR	Photovoltaic	Electrolyte	BPM	STF_CO2_	STF		
SGC	a-Ag	GaInP/GaInAs/Ge	0.4 M NaH_2_PO_4_ + 0.6 M Na_2_SO_4_ + NaOH	No	13.9%	15.6%	19 h	This work
IrO_2_	Au	perovskite PV	0.5 M NaHCO_3_	No	6.5%	7%	18 h	^4^
IrO_2_	CuAg	silicon PV	0.5 M CsHCO_3_	No	2%	3.2%	6 h	^5^
IrO_2_	CuAg	III–V/silicon tandem cells	0.5 M CsHCO_3_	No	5.6%	8.4%	−	^5^
IrO_2_	polymetric Ru complex	SiGe triple-junction	0.1 M phosphate buffer solution	No	4.6%	−	6 h	^6^
Co_3_O_4_	NiN-GS	GaInP_2_/GaAs/Ge	0.5 M KHCO_3_	No		∼10%	10 h*	^7^
SnO_2_–CuO	SnO_2_-CuO	GaInP/GaInAs/Ge	0.1 M CsHCO_3_| 0.25 M CsOH	Yes	12.4%	13.8%	5 h	^8^
Ni**	Zn	silicon PV**	0.5 M KHCO_3_| 1 M KOH	Yes	0.7%	4.6%	3 h	^9^
Ni**	Pd/C coated Ti	GaAs/InGaP/TiO_2_**	2.8 M KHCO_3_| 1 M KOH	Yes	10%	−	3 h	^10^
IrO_2_	Ag	ideal triple junction***	−	−	6.95%		−	^11^

*: refresh the electrolyte every few hours; **: integrated photoanode; ***: maximum achievable efficiency for CO generation from theoretical estimation using IrO_2_ for OER and silver for CO_2_RR

## Discussion

While developing high-performed OER catalysts in neutral solution has become a hurdle in realizing a practical CO_2_-reduction system, our results suggested the critical role of controlling the stability of catalyst in neutral conditions to achieve a highly active and sustainable oxygen evolution. In addition to Sr_2_GaCoO_5_ and Sr_2_AlCoO_5_, we screened several other A_2_BB’O_5_ type Brownmillerite oxides with A=Ca or Sr, B=Mn, Fe or Co, and B’=Ga or Al for oxygen evolution without attempting to optimize the recorded performance. The results summarized in Supplementary Table 8 suggested good potential of Brownmillerite oxides to catalyze oxygen-related reactions. Because the current work studied the activity of as-synthesized stoichiometric Sr_2_GaCoO_5_ crystallized in micron-sized particles (Supplementary Fig. 22), it is optimistic to expect further improving activity through strategies established from other perovskite family of catalysts^46^. In addition, the discovery of SGC as a highly active neutral-pH OER catalyst enables the pairing with various catalysts for efficient BPM-free CO_2_ reduction (Supplementary Note 1). Examples to generate possible products such as formate, hydrocarbon and oxygenate are assessed in the Supplementary Figure 23. The examination of these devices is therefore of great interest towards the realization of practical sunlight-driven CO_2_ reduction systems.

## Methods

### Density functional theory (DFT) calculations

DFT calculations were performed with the Vienna ab initio Simulation Package (VASP) using projector-augmented waves (PAW) pseudopotentials and the exchange-correlation functionals parametrized by Perdew, Burke, and Ernzerhof for the generalized gradient approximation (GGA)^47–49^. Numerical convergence to about 2 meV per formula unit was ensured by using a cutoff energy 520.0 eV and appropriate Gamma centered k-point meshes. The electronic structure was calculated using GGA+U method with the U value set as 3.3 for Co. The adsorption of OER intermediates (OH, O, and OOH) was evaluated on the Co-exposed (010) plane of Sr_2_GaCoO_5_ and Sr_2_AlCoO_5_. To mitigate the interaction between periodic images, a vacuum layer of 15 Å thickness was applied in the supercell.

### Materials synthesis and characterization

To synthesize Sr_2_GaCoO_5_, stoichiometric amounts of SrCO_3_, Ga_2_O_3_, and Co_3_O_4_ (all 99.9%, from Sigma Aldrich) were ball milled together and placed in an alumina crucible for the calcination at 1100 °C for 24 h. The mixtures were carefully ground in a mortar, followed by sintering at 1100 °C for 216 h with 3 intermediate re-grounding. The as-synthesized product was then ball-milled. The synthesis of Sr_2_AlCoO_5_ followed the same procedure using stoichiometric amount of SrCO_3_, Al_2_O_3_ and Co_3_O_4_ as the precursors and the sintering was carried out at 1250 °C for total 288 h with 4 intermediate re-grounding. The X-ray diffraction was collected using a Rigaku Smart Lab diffractometer equipped with a Cu K_α_ radiation (λ = 1.54178 Å) source at a scan rate of 0.1°/minute. Rietveld refinement of the XRD data was performed using the GSAS/EXPGUI package. X-ray photoelectron spectroscopy (XPS) spectra were collected with a PHI 5000 VersaProbe II X-ray photoelectron spectrometer using an Al K_α_ source. The X-ray parameter conditions were 15 kV, 25 W, pass energy of 23.5 eV and at a resolution of 0.2 eV/step. The sample was mounted on double-sided carbon tape and tilted at 45 degrees. An alternating Ar^+^ ion source was used for sputtering at 1 kV. The HRTEM study was performed using a JEM-2200FS high-resolution transmission electronic microscope with an in-column energy filter operated at 200 kV. The energy dispersive spectroscopy was taken with the Oxford INCA system at the energy resolution of 140 eV. High-angle annular dark-field scanning transmission electron microscopy imaging was carried out on a FEI Titan80–300 S/TEM microscope equipped with a probe spherical aberration corrector and the operation voltage was 300 kV. The collection angles of the annular dark-field detector for the inner and outer were 55 and 220 mrad, respectively. The magnetization measurements were performed using Magnetic Property Measurement System (MPMS) by Quantum Design.

### Oxygen evolution reaction

To prepare the electrode for oxygen evolution, a catalyst ink was first prepared by sonicating a mixture of active catalyst (Sr_2_GaCoO_5_ after ball milling or commercial IrO_2_ purchased from Alfa Aesar, 99.99%), acid-treated carbon black (CB, Alfa Aesar), Na^+^-exchanged Nafion® solution (5 wt%, Ion Power) in tetrahydofuran (THF, Sigma-Aldrich), and then drop-casted (10 μL) onto pre-polished glassy carbon disk electrodes (5 mm in diameter, Pine Instruments)^42^. The catalyst film was dried at room temperature for 12 h and the final composition of the film was 250, 50, and 50 µg/cm^2^ for the catalyst, CB, and Nafion®, respectively.

The OER experiments in neutral pH used the CO_2_-saturated solution of 0.4 M NaH_2_PO_4_ and 0.6 M Na_2_SO_4_ and proper amount of NaOH that tuned the pH to 7.0. The tests were performed at room temperature in three-electrode cells using a VSP multichannel potentiostat (Bio-logic). The counter electrode was a Pt coil isolated from the main electrochemical cell using a fritted glass tube. The working electrodes rotated at 1600 rpm using a rotating disc electrode system (Pine Instruments). Cyclic voltammetry (CV) was reported at a scan rate of 10 mV/s while linear sweep voltammogram (LSV) was performed at 1 mV/s. The potentials were measured versus a Ag/AgCl (4 M KCl) reference electrode. Except otherwise mentioned, all the potentials reported in the paper was corrected for IR drop and converted to the reference hydrogen electrode (RHE). The current density was evaluated based on the BET surface area of the catalyst

1 $$j_{BET} = \frac{I}{A} = \frac{I}{{mS_{BET}}}$$

where *I* is the measured current, *A* is the total surface area of the catalyst, m is the amount of catalyst on the electrode and *S*_*BET*_ is the BET surface area. The BET current density can be converted to the geometric current density

2 $$j_{geo} = j_{BET}\frac{{mS_{BET}}}{{A_{geo}}}$$

where *A*_*geo*_ is the geometric area of the electrode.

### Sunlight-driven CO_2_ reduction

To prepare the Sr_2_GaCoO_5_ anode for the test of CO_2_ reduction, a catalyst ink was prepared in the same way as described above and was subsequently drop-coated onto a carbon paper (FuelCellStore). The total apparent electrode size was 1.1 cm^2^ and the loading of Sr_2_GaCoO_5_ was 1 mg·cm^−2^. The anodized Ag electrodes were prepared from a silver foil (Alfa Aesar, 99.998%, 0.25 mm in thickness) polished and sonicated with deionized water before anodization. The anodization was conducted was carried out at 0.6 V potential for a total charge of 5.5 coulombs using platinum counter electrode, Ag/AgCl reference electrode and 0.1 M NaNO_3_ electrolyte. The apparent electrode sizes for the anode and cathode were the same.

Sunlight-driven CO_2_ reduction experiments were carried out in a prototype cell integrating a triple-junction GaInP/GaInAs/Ge photovoltaic cell (SpectroLab) and a two-chamber electrolyzer. The two electrodes were separated by a Nafion®117 membrane and only the product gases from a-Ag electrode was collected. The area of the photovoltaic cell was 0.555 × 0.555 cm (0.308 cm^2^). Before the electrochemical test, the PV characteristics of the photovoltaic cell was measured. The short circuit current was 0.0041 A. The current and voltage at the maximum power point was 0.0039 A and 2.255 V, respectively, corresponding to the maximum efficiency of 28.5% under 1 sun irradiation. The electrolytes were first saturated with CO_2_ for 30 min before the electrolysis. Gas phase product was sampled from the headspace of the cathode cell chamber using a gas-tight syringe (Hamilton) periodically every half an hour. A Thermo Scientific Trace GC Ultra gas chromatograph equipped with a VICI pulsed discharge detector and SUPELCO Carboxen^TM^ 1010 PLOT column was used to determine the amount of H_2_ and CO in the product. Ultra-pure (>99.999%) helium was used as carrier gas.

## Supplementary information

Supplementary Information

Peer review

## Data Availability

The data that support the findings of this study are available from the corresponding author upon request.
