# Supplementary material for: A high-performance oxygen evolution catalyst in neutral-pH for sunlight-driven CO2 reduction
Source: Nat Commun. 2019 Sep 9;10:4081. doi: 10.1038/s41467-019-12009-8 (PMC6733866; doi:10.1038/s41467-019-12009-8)
Supplement: Supplementary file 1 — Supplementary Information [file 41467_2019_12009_MOESM1_ESM.pdf]

Supplementary Information

for

A high-performance oxygen evolution catalyst in neutral-pH for sunlight-driven CO_2 reduction

Li Qin Zhou,[1] Chen Ling,[1*] Hui Zhou,[2] Xiang Wang,[3] Joseph Liao,[4], Gunugunuri K. Reddy,[1] Liangzi Deng,[5], Torin C. Peck,[1] Ruigang Zhang,[1] M. Stanley Whittingham,[2] Chongmin Wang,[6] Ching-Wu Chu,[5,7], Yan Yao[8] and Hongfei Jia[1]

[1] Toyota Research Institute of North America, Ann Arbor, MI, 48105, United States.
[2] Chemistry and Materials, Binghamton University, Binghamton, NY, 13902, United States
[3]Department of Mechanical Engineering and Materials Science, University of Pittsburgh, Pittsburgh, PA, 15261, United States
[4]Enli Technology Co. Ltd., Kaohsiung City, 82151, Taiwan
[5]Texas Center for Superconductivity and Department of Physics, University of Houston, Houston, TX, 77204, United States
[6]Environmental Molecular Sciences Laboratory, Pacific Northwest National Laboratory, Richland, WA, 99352, United States
[7] Lawrence Berkeley National Laboratory, Berkeley, CA, 94720, United States
[8]Department of Electrical and Computer Engineering and Texas Center for Superconductivity (TcSUH), University of Houston, Houston, TX, 77204, United States.

* Corresponds and requests for materials should be addressed to C. L. (chen.ling@toyota.com).

Supplementary Figures

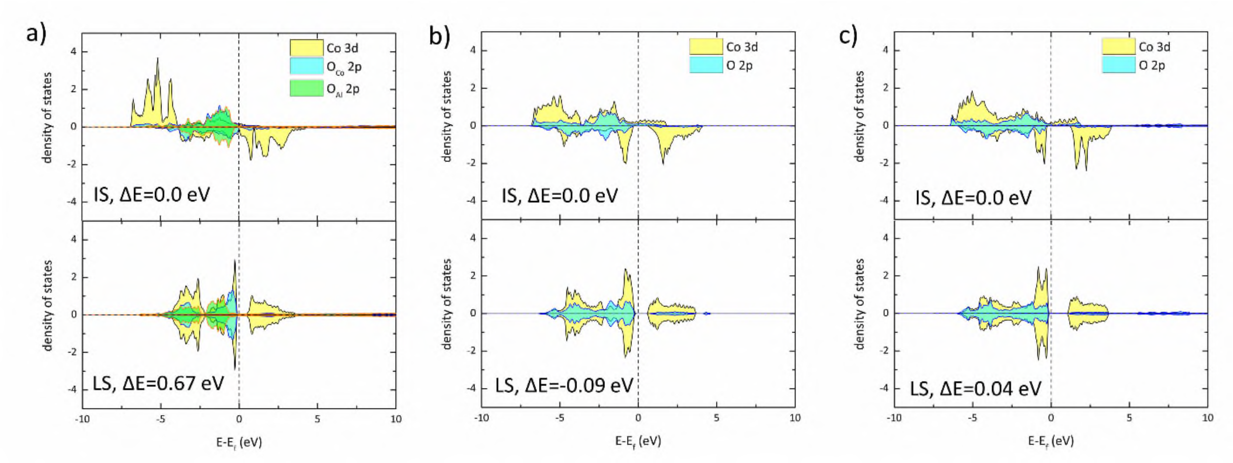

Supplementary Figure 1. Electronic structures of cobalt-containing Brownmillerite and perovskite compounds with Co^{3+} staying at intermediate spin (IS) and low spin (LS) state. **a,** Brownmillerite Sr_2AlCoO_5 (SAC). **b,** perovskite $LaCoO_3$ (LCO), **c,** perovskite $YCoO_3$ (YCO). The energy difference between different spin states for the perovskite compounds are generally less. For LCO the ground states at 0 K is low spin and it transits to higher spin states above 100 K. For YCO the calculation shows the IS state slightly favors the LS state. For SAC, the IS state is strongly more stable than the LS state, similar to the results for Brownmillerite Sr_2GaCoO_5.

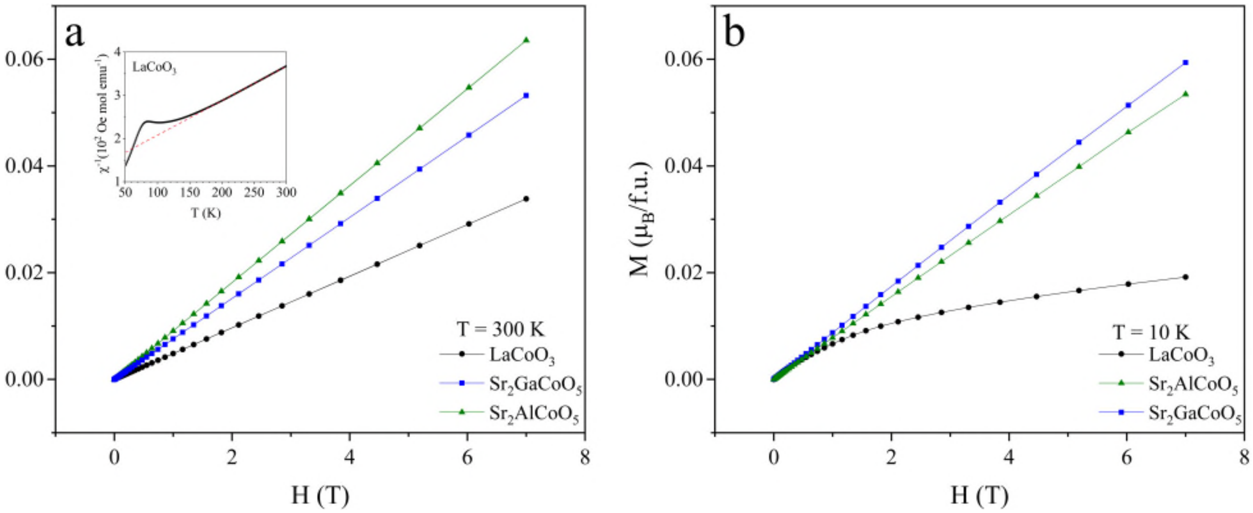

Supplementary Figure 2. Magnetic Behaviors of perovskite $LaCO_3$, Brownmillerite Sr_2AlCoO_5 and Sr_2GaCoO_5. Single cobalt magnetic moment depends on the magnetic field at the temperature of **a,** 300K and **b,** 10 K. The M-H curves demonstrate that the cobalt magnetic moment of Sr_2GaCoO_5 and Sr_2AlCoO_5 have larger values than $LaCoO_3$, indicating the Co^{3+} ions in Sr_2GaCoO_5 and Sr_2AlCoO_5 have a higher ratio of high spin (HS) state/low spin (LS) state than $LaCoO_3$. The insert showed the temperature dependence inverse susceptibility for the $LaCoO_3$ sample under H = 0.1 T. The red dash line represents the Curie-Weiss fitting result. The calculated μ_{eff} is around 3.16 μ_B and the spin sates are estimated to be 42% HS + 58% LS for Co^{3+} ions in $LaCoO_3$.

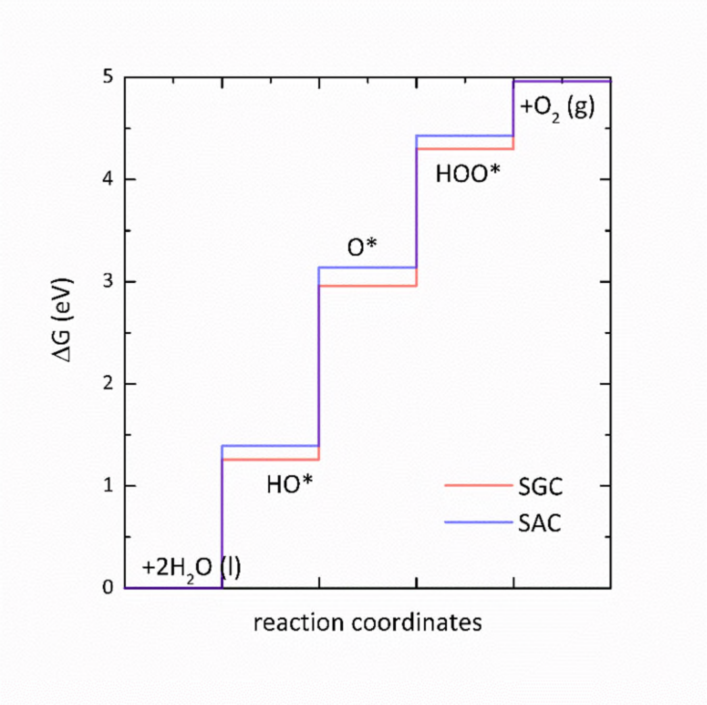

Supplementary Figure 3. Energy diagram for oxygen evolution on Co-terminated (010) surface of Sr_2SrCoO_5 and Sr_2AlCoO_5. For both SGC and SAC, the highest energy barrier is to convert HO* to O* (Supplementary Equation 2), attributed to the formation of unstable oxygen radicals due to the strongly covalent Co^{4+}-O bond.[1]

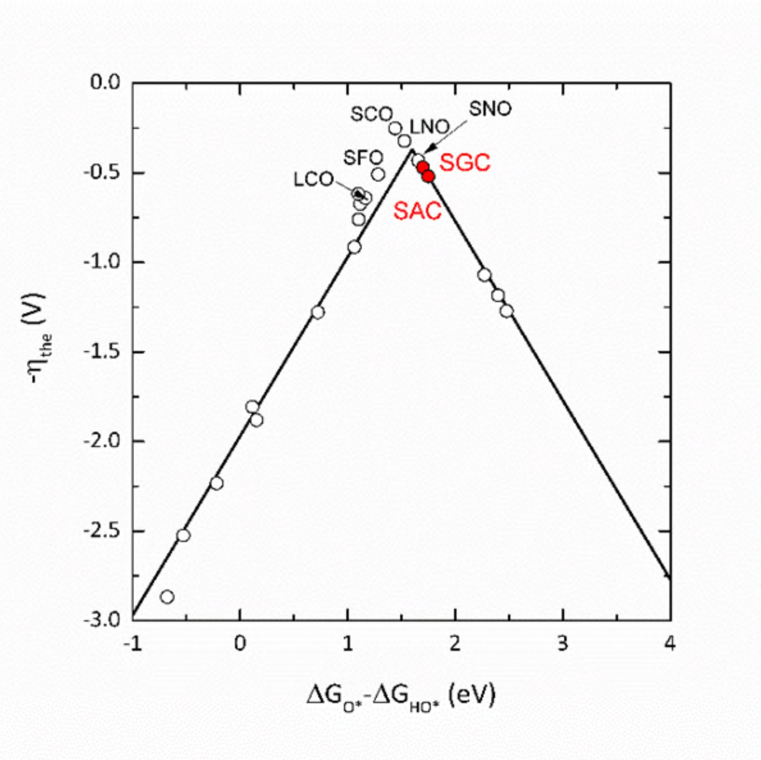

Supplementary Figure 4. Theoretical overpotential calculated as the largest barrier to accomplish Supplementary Equation 1-4. The solid line shows the volcano curve established by the relation between ΔG_{OOH} and ΔG_{OH} as ΔG_{OOH}+ ΔG_{OH}=3.2 eV. The open symbols were taken from the work of Man et al.[2]

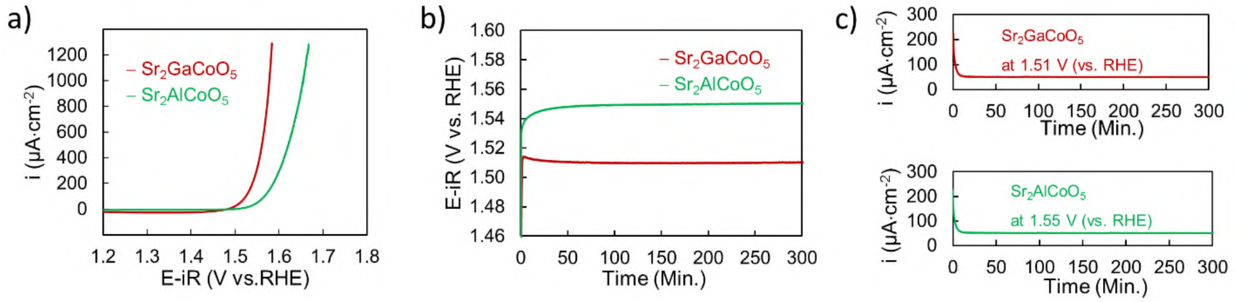

Supplementary Figure 5. Characterization of the Sr_2GaCoO_5 and Sr_2AlCoO_5 OER specific activity at pH 13. a, Linear sweep voltammograms. **b,** Galvanostatic experiments at 50 µA/cm^2**. c,** Potentiostatic experiments at 1.51 V vs. RHE for Sr_2GaCoO_5 and 1.55 V vs. RHE for Sr_2AlCoO_5.

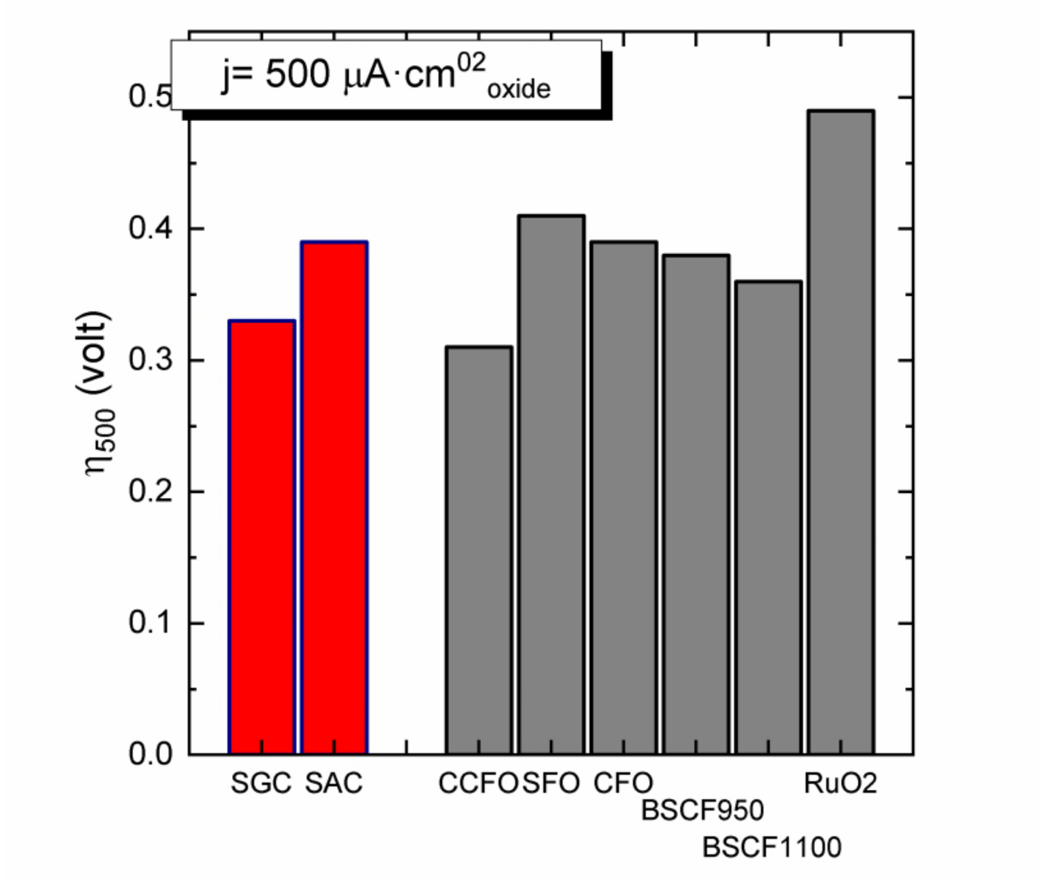

Supplementary Figure 6. The overpotential of Brownmillerite Sr_2GaCoO_5 and Sr_2AlCoO_5 at the current density of 500 μA·cm_{oxide}^{-2} at pH 13 (0.1 M KOH). This current density roughly corresponded to the geometric current density of 10.5 mA·cm_{geo}^{-2} for SGC, hence quantifying the performance at high current densities in potentially practical conditions. The state-of-art catalysts used for comparison included $CaCu_3Fe_4O_{12}$ (CCFO), $CaFeO_3$ (CFO), $SrFeO_3$ (SFO), $Ba_{0.5}Sr_{0.5}Co_{0.8}Fe_{0.2}O_{3-\delta}$ calcined at 950 and 1,100 °C (BSCF950 and BSCF1100, respectively) and RuO_2. All the reference data were taken from the work of Yagi et al.[3] The η_{500} of SGC is only 0.02 V higher than that of CCFO, and considerably lower than other oxide catalysts including two BSCF samples. The η_{500} of SAC was 0.06 V higher than that of SGC, but still comparable to BSCF and CFO.

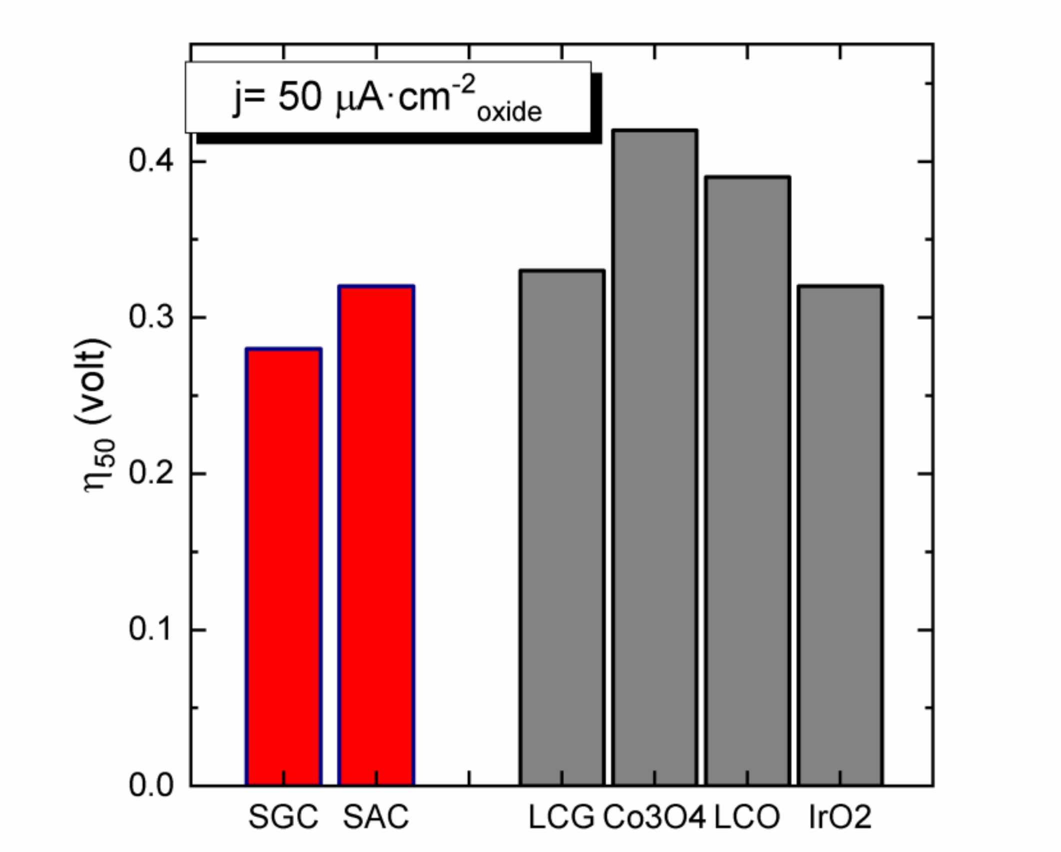

Supplementary Figure 7. The overpotential of Brownmillerite Sr_2GaCoO_5 and Sr_2AlCoO_5 at the current density of 50 μA·cm_{oxide}^{-2} at pH 13 (0.1 M KOH). This low current further minimized the influence of extra resistance caused by mass transportation, thus providing information about the intrinsic OER activity. Several Co-based OER catalysts were used for comparison, including Co_3O_4,[4] $LaCoO_3$ (LCO),[1,5] Li_2CoGeO_4 (LCG),[4] and IrO_2.[5] While it is not surprising to see that the OER activity SGC and SAC was significantly better than those of typical benchmark catalysts such as Co_3O_4 and LCO, the η_{50} for SGC and SAC was 0.04 and 0.01 V lower than that of LCG, respectively.

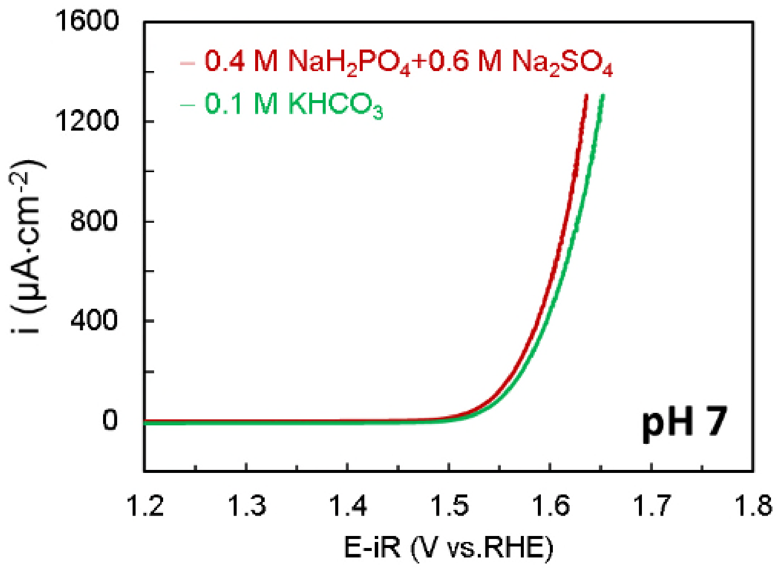

Supplementary Figure 8. Linear sweep voltammogram of Sr_2GaCoO_5 in different pH 7.0 electrolytes. For the $KHCO_3$ electrolyte the solution is purged with a mixture of CO_2 and N_2, since purging with pure CO_2 lowers the pH to 6.8. The effect of the electrolyte on the OER performance of SGC is marginal, with the difference of overpotential generally less than 0.01 V. This slight difference can be partially attributed to the different concentration of ions in the electrolytes.

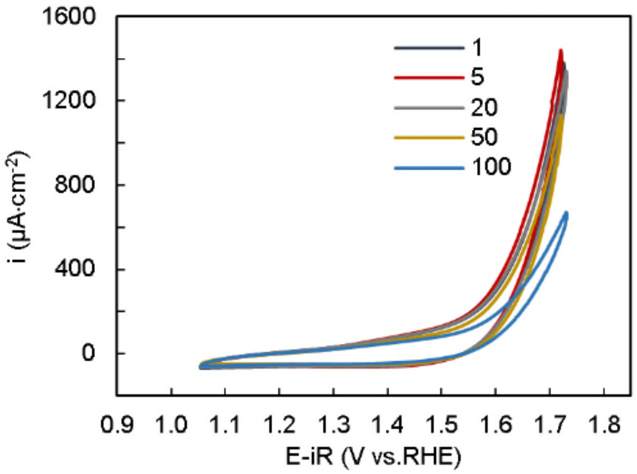

Supplementary Figure 9. Cyclic voltammetry of Sr_2AlCoO_5 for oxygen evolution at pH 7. The cycling number is shown in the legend. The current density showed a gradual but obvious decrease in the cycling. At 1.7 V vs RHE, the current dropped by more than 50% in 100 cycles.

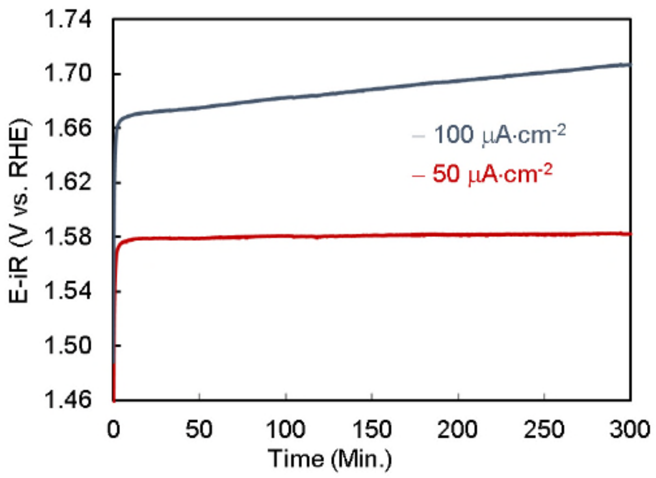

Supplementary Figure 10. Galvanostatic test of Sr_2AlCoO_5 for oxygen evolution at pH 7. The voltage only slightly changed when the constant current density was held at 50 $\mu A \cdot cm_{oxide}^{-2}$ for five hours. At the current density of 100 $\mu A \cdot cm_{oxide}^{-2}$, an apparent increase of voltage was observed in the measurements.

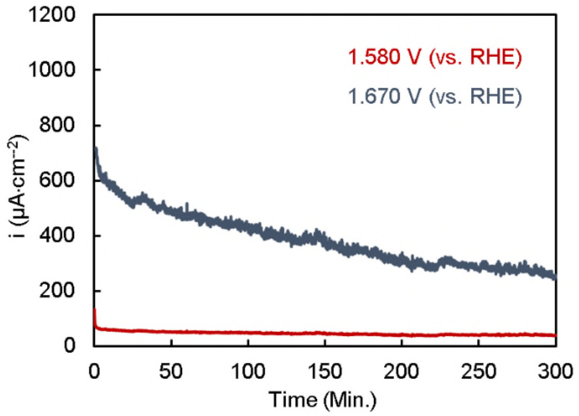

Supplementary Figure 11. Potentiostatic test of Sr_2AlCoO_5 for oxygen evolution at pH 7. At 1.58 V vs RHE the current density stabilized at about 50 $\mu A \cdot cm_{oxide}^{-2}$ for five hours, while at 1.67 V vs RHE the current density rapidly deceased in the measurement.

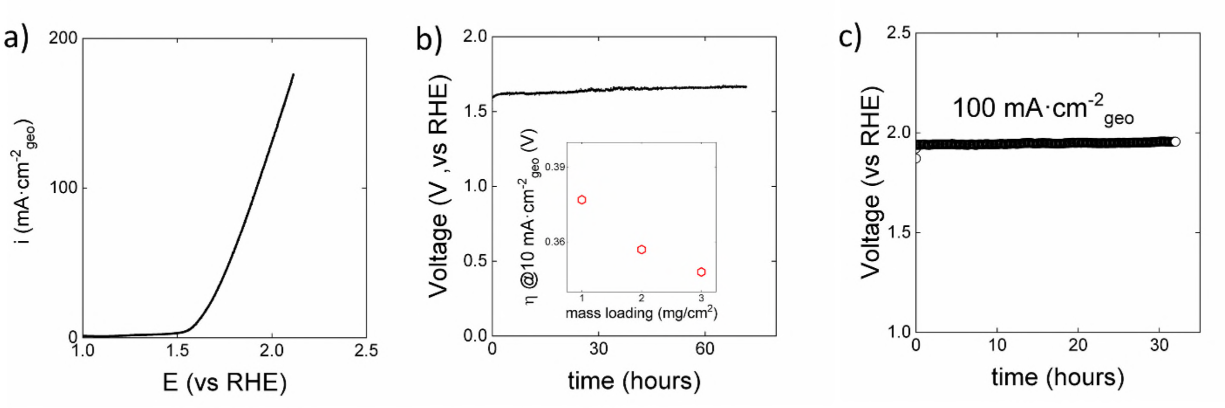

Supplementary Figure 12. Performance of a carbon paper electrode loaded with Sr_2GaCoO_5 for oxygen evolution reaction in neutral pH. **a,** Linear sweep voltammogram of the carbon paper electrode. The loading of catalyst was 1 mg·cm^{-2}. **b,** Overpotential of the carbon paper electrode for 72 hours of operation at 10 mA·cm^{-2}. The overpotential was 0.377 V and increased by less than 1 mV per hour. The insertion showed the measured overpotential at the same geometric current density for different loadings. **c,** Voltages of the carbon paper electrode at 100 mA. The loading of catalyst was 1 mg·cm^{-2}. At high current densities the effect of resistant increases. At 100 mA·cm^{-2}, the measured resistance caused 0.245 V loss of voltage for a 0.5 cm^2 electrode (R=4.9 Ω in LSV measurement and 4.8 Ω in galvanostatic measurements). Note at such high voltages the carbon electrode may be vulnerable for oxidation.

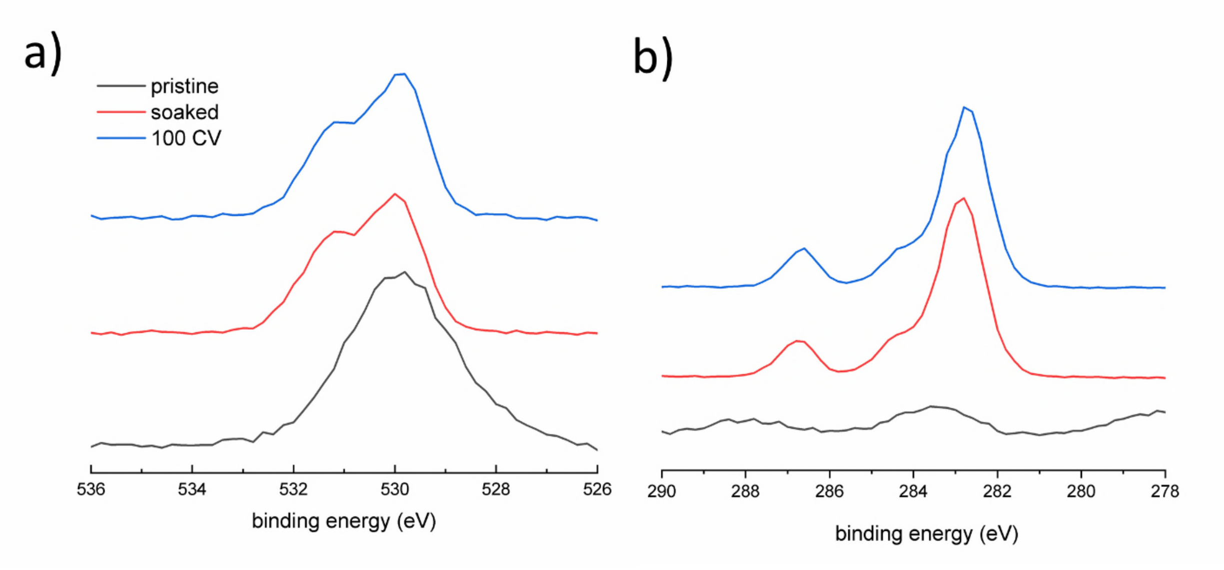

Supplementary Figure 13. X-ray spectroscopy results for Sr_2GaCoO_5. a, oxygen 1s and **b,** and carbon 1s signals**.** The data was taken on as-prepared samples without Ar^+ etching. Compared to the pristine material, the contact of electrolyte was signaled by the more adsorption of hydroxyl group at the peak of 531.2 eV on oxygen 1s spectra. The weak signal of carbon spectra for the pristine material can be attributed to the atmospheric CO_2 adsorbed on the surface. For the soaked and cycled samples, the strong carbon signal comes from the carbon-containing component in the catalyst ink, such as carbon black and Nafion.

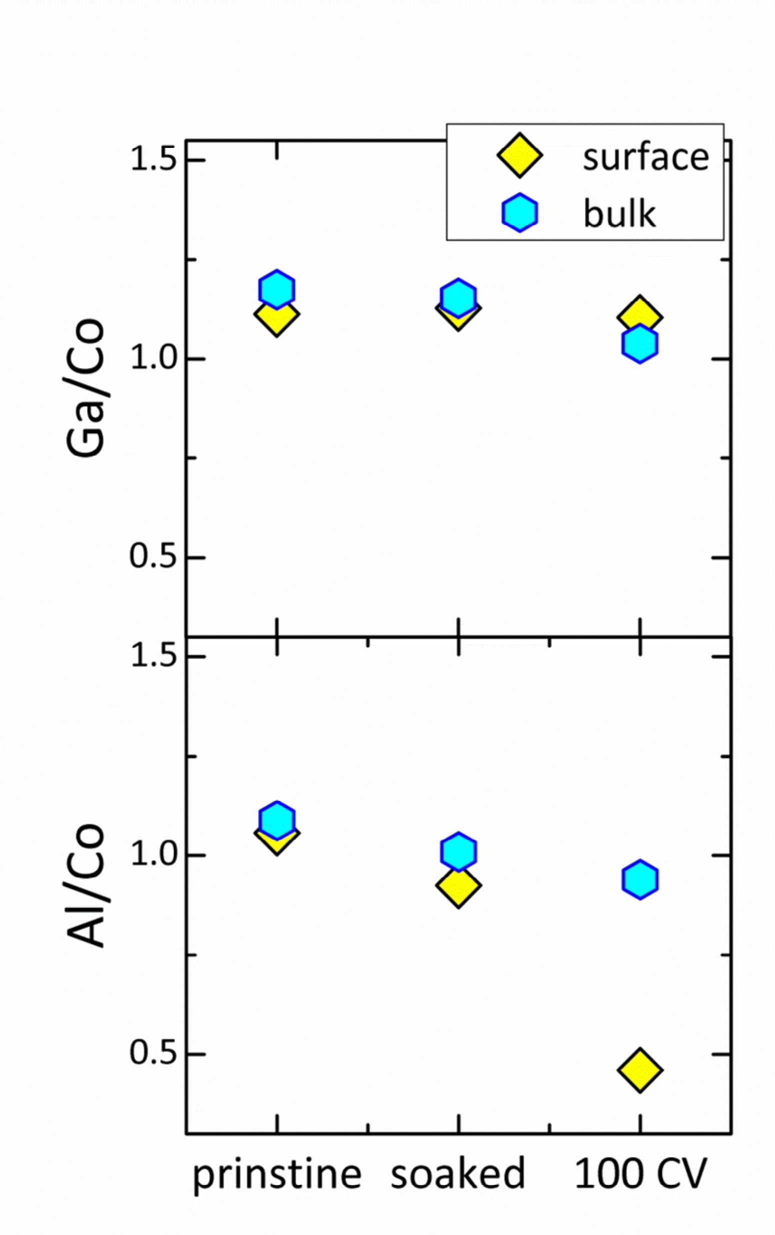

Supplementary Figure 14. Metal ratio in the surface and bulk of Sr_2GaCoO_5 and Sr_2AlCoO_5. The estimation is performed by integrating area of XPS 2p peaks normalized by the ASF factor, 0.234 for Al, 3.59 for Co and 3.72 for Ga.

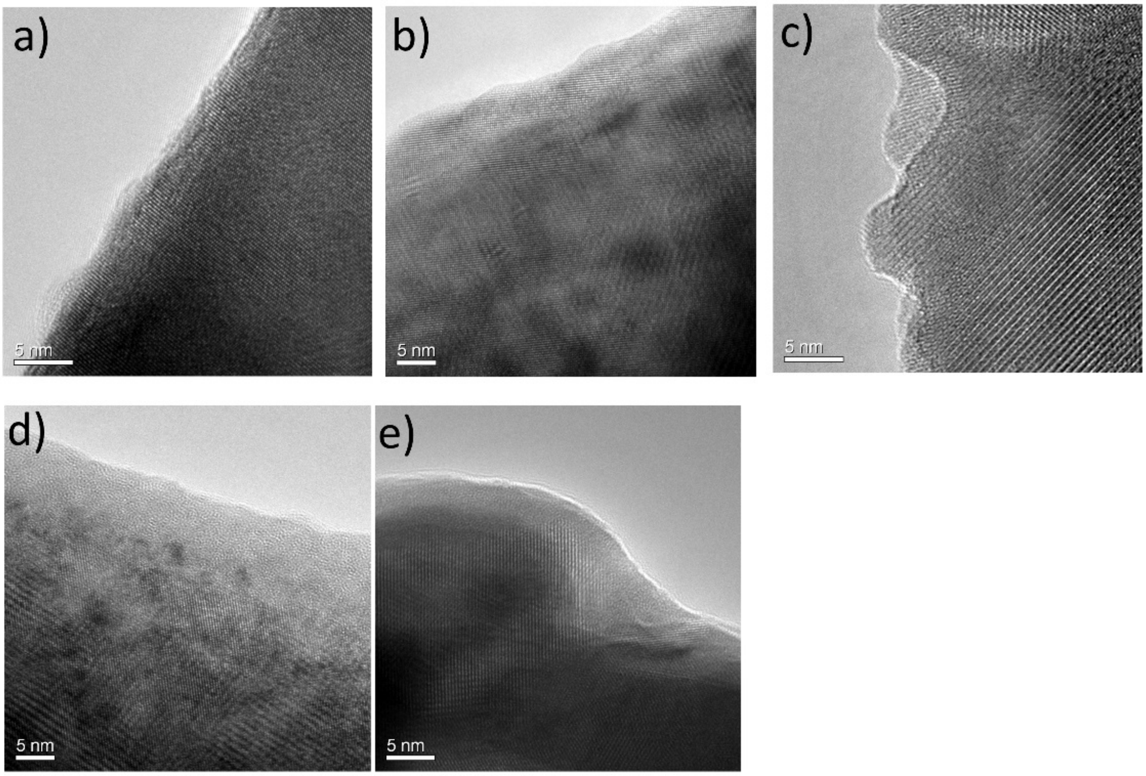

Supplementary Figure 15. HRTEM images of Sr_2GaCoO_5 and Sr_2AlCoO_5 after oxygen evolution operation in neutral pH. a to c, Sr_2GaCoO_5; **d to e,** Sr_2AlCoO_5.

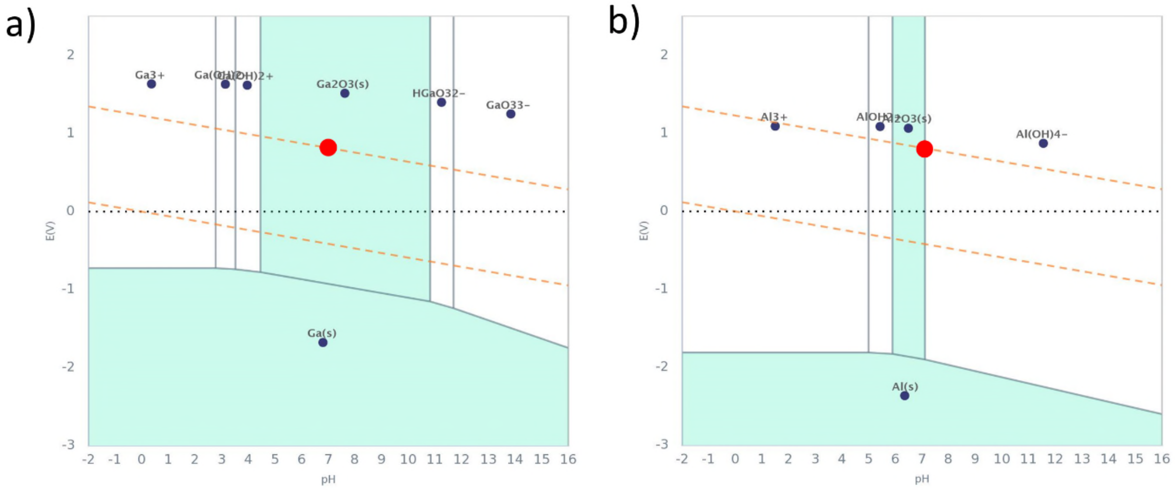

Supplementary Figure 16. Computational Pourbaix diagram. a, Ga-O-H and **b,** Al-O-H systems. The red dot shows the position of oxygen evolution at zero overpotential at pH 7. Regions with the existence of stable solid is filled with cyan color. The figure is generated by Materials Project,[6-8] assuming the cation concentration in the solution is 10^{-8} M. The comparison of Ga-O-H and Al-O-H Pourbaix diagram showed more stable solid gallium oxide than aluminum oxide near neutral pH. Aluminum oxide dissolves to form $Al(OH)_4^-$ at pH slightly higher than 7, while the stable range of gallium oxide is much wider.

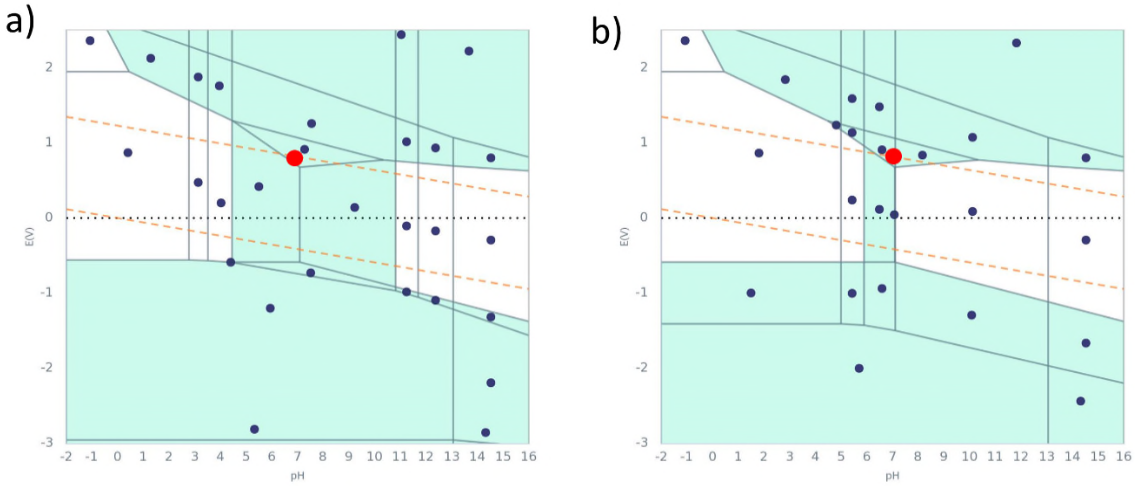

Supplementary Figure 17. Computational Pourbaix diagram. a, Ga-Sr-Co-O-H and **b,** Al-Sr-Co-O-H systems. The red dot shows the position of oxygen evolution at zero overpotential at pH 7. Regions with the existence of stable solid is filled with cyan color. While the general features of more complicated Pourbaix diagrams of Ga-Sr-Co-O-H and Al-Sr-Co-O-H systems are similar, the stability range of solid gallium oxide is also noticeably wider than that of solid aluminum oxide near neutral pH, the same as the characteristics of Ga-O-H and Al-O-H systems.

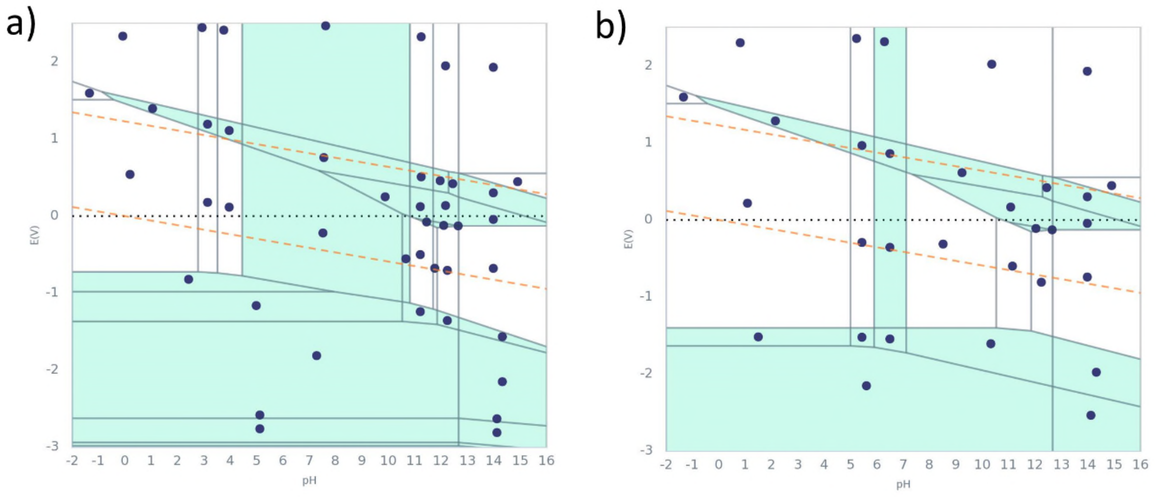

Supplementary Figure 18. Computational Pourbaix diagram. a, Ga-Sr-Mn-O-H and **b,** Al-Sr-Mn-O-H systems. Regions with the existence of stable solid is filled with cyan color. Similar to the results in Supplementary Figure 15 and 16, the Ga system has a much wider range of stability near neutral pH than the Al system, indicating that the stability of SGC and SAC can be related to the solubility of gallium oxide and aluminum oxide under OER conditions

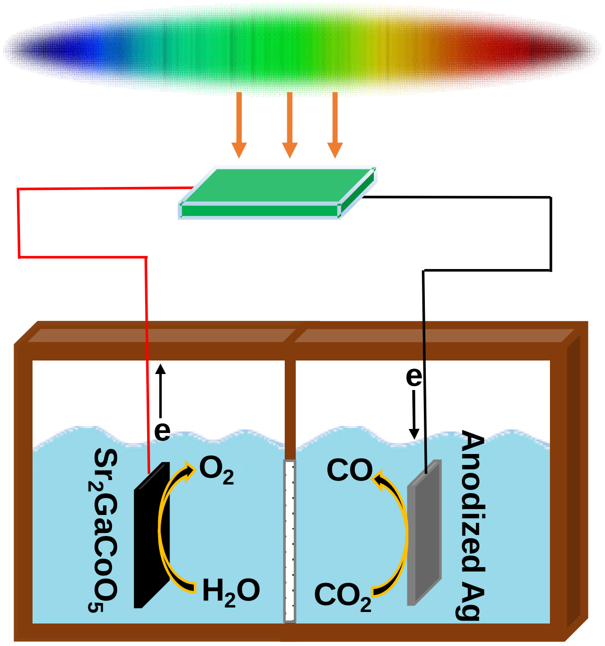

Supplementary Figure 19. Schematic of the integrated sunlight-driven CO_2 reduction system. The system consisted of a photovoltaic, and a two-chamber electrolyzer with Sr_2GaCoO_5 as water oxidation (anode) and anodized Ag plate as CO_2 reduction (cathode) catalysts. The two chambers were separated by a Nafion®117 membrane. The geometric areas of the electrodes are 1 cm^2.

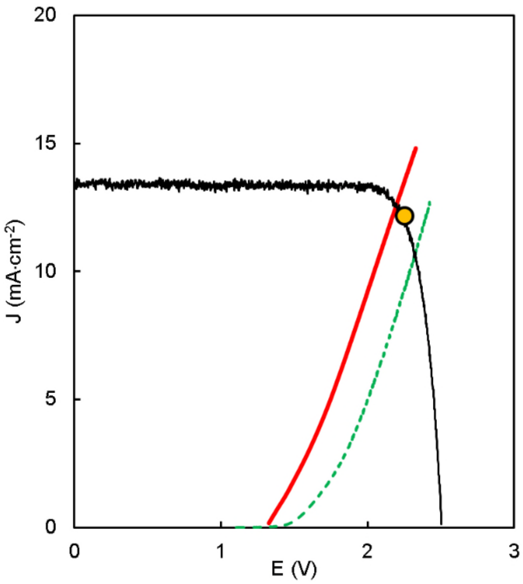

Supplementary Figure 20. Current density-voltage characteristics of individual components in sunlight-driven CO_2 reduction device. The black curve shows the photovoltaic J-V curve. The red and green curves show the electrochemical J-V curve using Sr_2GaCoO_5 and IrO_2 for oxygen evolution catalyst, respectively. The orange point marks the maximum power point of the solar cell.

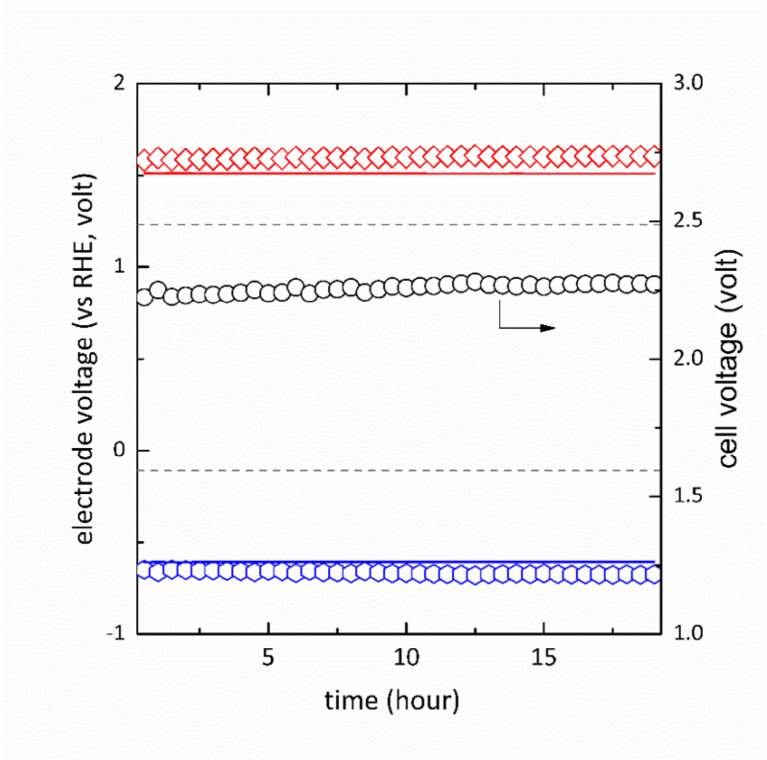

Supplementary Figure 21. Potentials on the Sr_2GaCoO_5 cathode and anodized Ag anode during 19 hours of sunlight-driven CO_2 reduction. The potential is monitored with Ag/AgCl reference electrode. The cathode potential is shown in red and the anode potential is shown in blue. The solid line shows the potential estimated from the Tafel relation using the measured current density. The measured voltage differed with the Tafel estimation by 0.06 V and 0.08 V on the cathode and anode, respectively. The difference between the real potential and the estimated potential is attributed to (i) iR correction and (ii) the mass transport resistance. The cell voltage is shown in black circles and the grey dotted line shows the position of equilibrium potential for oxygen evolution and CO_2 reduction to CO at pH 7.

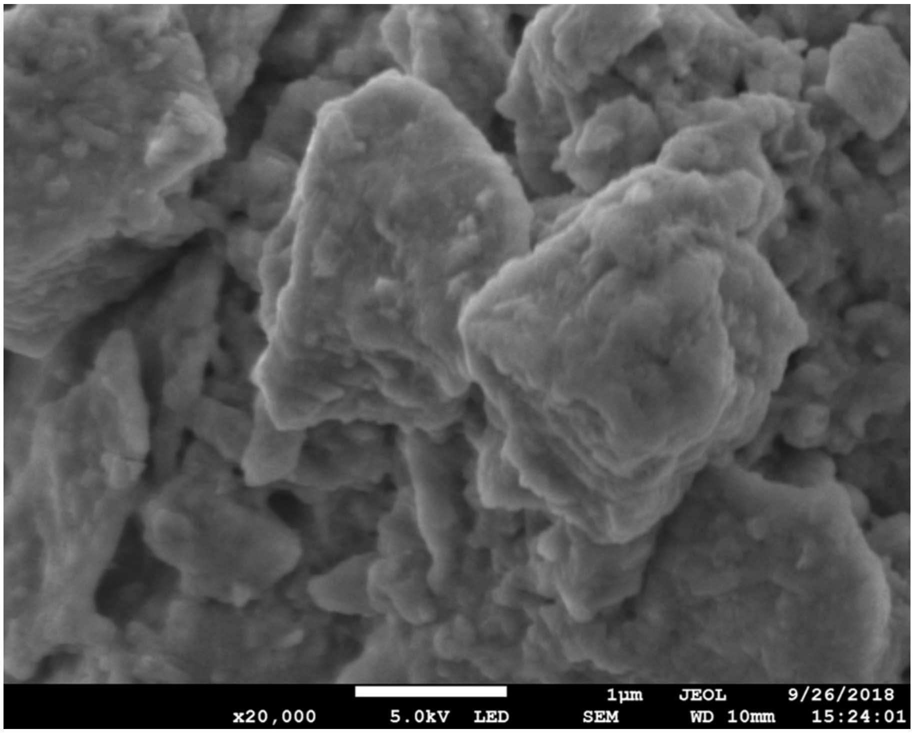

Supplementary Figure 22. Morphology of Sr_2GaCoO_5 from scanning electron spectroscopy imaging.

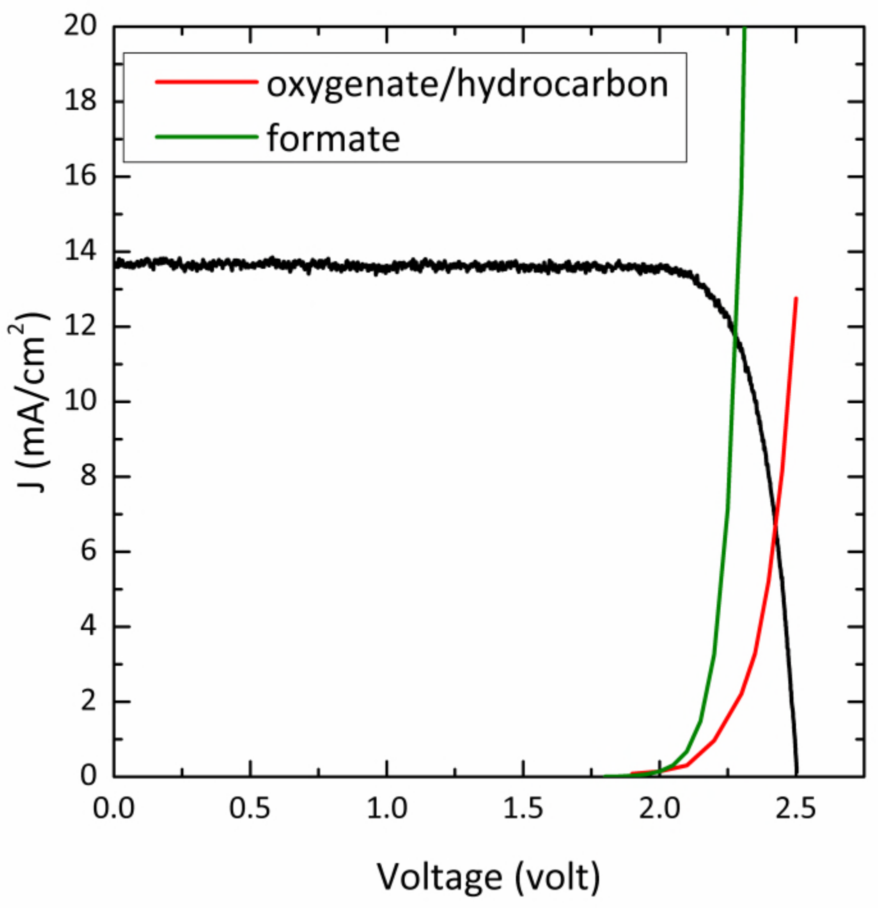

Supplementary Figure 23. Estimation of the pairing of Sr_2GaCoO_5 cathode and two CO_2 reduction anodes for sunlight-driven CO_2 reduction, CuAg nanocoral for the generation of oxygenate/hydrocarbon and sulfur modified tin for the generation of formate. The solar-to-fuel efficiency is calculated as $STF = VJ/100$, where V is the equilibrium voltage for CH_3OH and HCOOH production, 1.19 V and 1.42 V, respectively, and the factor of 100 is the power of 1 sun irradiation (100 mW·cm^{-2}). Assuming a Faradaic efficiency of 93% for both products, the STF efficiency is 15.4% for formate and 7.5% for oxygenate/hydrocarbon. Both values are significantly higher than the start-of-art level in the literatures, 10% for formate production and

5.6% for oxygenate/hydrocarbon production.[9,10] The high STF efficiency for formate production is also partially related to the highly active Sn anode.[11]

Supplementary Tables

Supplementary Table 1. Refined crystallographic parameters for Sr_2GaCoO_5 from Rietveld refinement.

atom	x	y	z	Occupancy	U_{iso}
Sr	0.0163(1)	0.1126(0)	0.5	1.0000	8
Co1	0	0	0	1.0000	4
Co2	-0.0657(2)	0.25	-0.0449(5)	0.0025(8)	8
Ga	-0.0657(2)	0.25	-0.0449(5)	0.4975(8)	8
O1	0.25	-0.0176(3)	0.25	1.0000	8
O2	0.0382(8)	0.1419(2)	0	1.0000	8
O3	0.8928(13)	0.25	0.6196(13)	0.5000	8

R_{wp} = 9.73%, R_p = 7.3%, χ^2 = 1.294, a = 5.6226(1) Å, b = 15.7596(2) Å, c = 5.4625(1) Å, V = 484.04(2) Å^3, ρ_{calc} = 5.267 g/cm^3. The space group is Icmm.

Supplementary Table 2. Bulk and surface composition of Sr_2GaCoO_5 after 100 CV scans in neutral-pH.

M_{bulk} (%)			$M_{surface}$ (%)			ΔM (%)		
Sr	Ga	Co	Sr	Ga	Co	Sr	Ga	Co
53.3	22.1	23.6	58.4	21.1	20.5	5.1	-1	-3.1
55.2	21.8	21.7	60.2	21.9	17.9	5	0.1	-3.8
56.3	21.9	20.8	61.2	20.2	18.6	4.9	-1.7	-2.2
57.9	19.5	23.6	60.8	20.7	18.5	2.9	1.2	-5.1
56.2	22.8	21.2	59.9	19.6	20.5	3.7	-3.2	-0.7
53.6	24.1	21.3	58.5	22	19.5	4.9	-2.1	-1.8
56.3	19.3	24.4	60.2	19.4	20.4	3.9	0.1	-4
56.5	24.9	18.4	60.6	22.2	17.2	4.1	-2.7	-1.2
59.3	21.7	19.9	62.3	17.5	20.2	3	-4.2	0.3
55.6	24.3	20.4	59.2	21.8	19	3.6	-2.5	-1.4

Supplementary Table 3. Bulk and surface composition of Sr_2GaCoO_5 after soaking in neutral-pH for four hours.

M_{bulk} (%)			$M_{surface}$ (%)			ΔM (%)		
Sr	Ga	Co	Sr	Ga	Co	Sr	Ga	Co
52.3	23.5	24.2	54.3	22.7	23	2	-0.8	-1.2
49.3	24.9	25.8	53.8	24.1	22.1	4.5	-0.8	-3.7
48.3	25.5	26.2	51.9	24.7	23.4	3.6	-0.8	-2.8
50.3	26.4	23.3	52.8	25.6	21.6	2.5	-0.8	-1.7
50.5	22.3	27.2	55.8	21.5	22.7	5.3	-0.8	-4.5
48.6	27.9	23.4	54.1	27.5	18.4	5.5	-0.4	-5
51.3	24.7	24	54	23.9	22.1	2.7	-0.8	-1.9
48.5	27.3	24.2	50.2	26.5	23.3	1.7	-0.8	-0.9
51.6	25.9	22.5	55.1	25.8	19.1	3.5	-0.1	-3.4
52.4	27.3	20.3	54.1	26.5	19.4	1.7	-0.8	-0.9

Supplementary Table 4. p-value in the equivalence test to evaluate the effect of soaking and OER operation on the composition of Sr_2GaCoO_5. The null hypothesis was rejected at 95% confidence level for all three metal species.

	Sr	Ga	Co
p_a	4E-5	0.036	0.014
p_b	0.019	2E-5	0.002

Supplementary Table 5. Bulk and surface composition of Sr_2AlCoO_5 after 100 CV scans in neutral-pH.

M_{bulk} (%)			$M_{surface}$ (%)			ΔM (%)		
Sr	Al	Co	Sr	Al	Co	Sr	Al	Co
59.1	20.6	20.3	65.3	14.4	20.3	6.2	-6.2	0
56.2	20.4	23.4	62.2	15.9	21.9	6	-4.5	-1.5
53.2	20.5	26.3	59.1	13.8	27.1	5.9	-6.7	0.8
54.4	18.0	27.6	60.4	14.5	25.1	6	-3.5	-2.5
54.2	21.4	24.4	60.2	12.9	26.9	6	-8.5	2.5
55.3	22.7	22.0	61.3	15.3	23.4	6	-7.4	1.4
58.4	17.9	23.7	64.4	14.7	20.9	6	-3.2	-2.8
56.7	23.6	19.7	62.7	15.5	21.8	6	-8.1	2.1
56.3	20.3	23.4	62.3	10.8	26.9	6	-9.5	3.5
54.6	22.9	22.5	60.6	15.1	24.3	6	-7.8	1.8
56.0	20.4	23.6	62.0	13.8	24.2	6	-6.6	0.6
56.9	21.6	21.5	62.9	10.5	26.6	6	-11.1	5.1
57.3	21.1	21.6	63.3	12.9	23.8	6	-8.2	2.2
56.5	23.5	20.0	62.3	12.8	24.9	5.8	-10.7	4.9
54.3	22.0	23.7	60.5	13.1	26.4	6.2	-8.9	2.7

Supplementary Table 6. Bulk and surface composition of Sr_2AlCoO_5 after soaking in neutral-pH for four hours.

M_{bulk} (%)			$M_{surface}$ (%)			ΔM (%)		
Sr	Al	Co	Sr	Al	Co	Sr	Al	Co
52	26.2	21.8	55.9	24.8	19.3	3.9	-1.4	-2.5
53.2	24.4	22.4	54.8	23.1	22.1	1.6	-1.3	-0.3
48.7	23.8	27.5	51.6	23.8	24.6	2.9	0	-2.9
52.1	26.9	21	54.9	21.2	23.9	2.8	-5.7	2.9
54.1	25.4	20.5	51.7	24.8	23.5	-2.4	-0.6	3
53.3	23.1	23.6	53.6	18.2	28.2	0.3	-4.9	4.6
51.8	24.5	23.7	53.4	22.9	23.7	1.6	-1.6	0
49.2	25.1	25.7	51.5	22	26.5	2.3	-3.1	0.8
51.9	26	22.1	52.5	21.4	26.1	0.6	-4.6	4
52.1	21.7	26.2	56.4	20	23.6	4.3	-1.7	-2.6
50.1	27.8	22.1	53.7	22.1	24.2	3.6	-5.7	2.1
52.6	24.3	23.1	53.6	23.8	22.6	1	-0.5	-0.5
49.8	26.9	23.3	49.2	20.7	30.1	-0.6	-6.2	6.8
52.3	25.5	22.2	54.7	21.3	24	2.4	-4.2	1.8
53.9	26.9	19.2	53.7	23.1	23.2	-0.2	-3.8	4

Supplementary Table 7. p-value in the equivalence test to evaluate the effect of soaking and OER operation on the composition of Sr_2AlCoO_5. The equivalent test cannot reject the null hypothesis that Al and Sr content did not change after OER operation, suggesting the B'-leaching induced surface reconstruction of SAC during oxygen evolution, which commonly happened when the oxygen p-band is far below the Fermi level.[1]

	Sr	Al	Co
p_a	<1E-6	0.225	1E-5
p_b	0.115	<1E-6	9E-6

Supplementary Table 8. OER performance Brownmillerite oxides $A_2BB'O_5$ with A=Ca or Sr, B=Mn, Fe or Co, and B'=Ga or Al). The synthesis of these oxides was carried out in a solid solution route. In a typical procedure, the stoichiometric amounts of nitrate precursors of $A(NO_3)_2$, $B(NO_3)_3$ and $B'(NO_3)_3$ were dissolved separately in water and mixed together. 28% ammonia hydroxide was then added to the solution until the co-precipitation completed. The precipitate was kept at 250 °C for overnight to evaporate the water and excess ammonia, followed by calcination at 450 °C for 2 hours. After the primary calcination, the powder was pressed into pellets and the sintering was performed at 1250 °C for 24 hours. The product was then ball milled before analysis. The synthesis of Ca_2AlCoO_5, Sr_2AlMnO_5 and Ca_2GaCoO_5 was not successful, and for Ca_2GaMnO_5 (CGM) the final product contained Ca_2MnO_4 impurity as detected from XRD. The failure to synthesis these pure phases can be attributed to insufficient calcination. The pure phases of Ca_2AlMnO_5, Ca_2AlFeO_5 and Ca_2GaFeO_5 were successfully obtained and for Sr_2AlFeO_5 only minor impurity was contained in the product. The evaluation of OER performance for these Brownmillerite oxides was carried out in 0.1 M KOH solution (pH=13) using a rotating disk electrode. The overpotential was measured at the current density of 50 $\mu A \cdot cm_{oxide}^{-2}$.

	BET area ($m^2 \cdot g^{-1}$)	Note	OER overpotential (V)
Ca_2AlMnO_5	2.9	pure phase	0.46
Ca_2AlFeO_5	2.9	pure phase	0.41
Ca_2AlCoO_5	-	Unknown phase	
Sr_2AlMnO_5	-	Unknown phase	
Sr_2AlFeO_5	7.8	minor impurity	0.42
Ca_2GaMnO_5	3.0	contains Ca_2MnO_4	0.5
Ca_2GaFeO_5	3.7	pure phase	0.44
Ca_2GaCoO_5	-	Unknown phase	
Sr_2GaMnO_5	-	not synthesized	
Sr_2GaFeO_5	-	not synthesized	

Supplementary Methods

Computational hydrogen electrode method

To evaluate the catalytic OER activity on oxide surface, the OER mechanism is assumed to occur into four consecutive electron transfer steps using the computational hydrogen electrode method

$H_2O(l) + * \rightarrow H^+ + e^- + HO*$ (Supplementary Equation 1)

$HO* \rightarrow H^+ + e^- + O*$ (Supplementary Equation 2)

$H_2O(l) + O* \rightarrow H^+ + e^- + HOO*$ (Supplementary Equation 3)

$HOO* \rightarrow H^+ + e^- + O_2(g)$ (Supplementary Equation 4)

Here * denotes an adsorption site and A* denotes the adsorbed species A on the surface. Using the computational hydrogen electrode method,[2] the standard free energies of each step are derived from the calculated energies of adsorbed intermediates.

Statistical analysis of the surface composition

To examine the effect of OER operation on the surface composition, the surface and bulk composition of SGC particles were measured for samples soaked in the electrolyte for four hours and for samples after 100 CV scans using EDS. For each sample the measurements were carried out at ten random spots. The difference between the surface and the bulk composition ($\Delta M = M_{surface} - M_{bulk}$) was then statistically evaluated. We used the equivalence test (TOST) to examine the null hypothesis that the OER operation changed the surface composition so that a significant difference can be observed.[12] It can be formulated

$H_0: |\Delta M_{OER} - \Delta M_{OER}| \geq \theta$ (Supplementary Equation 5)

$H_1: |\Delta M_{OER} - \Delta M_{OER}| < \theta$ (Supplementary Equation 6)

The acceptance criterion θ was set to be the same as the average standard deviation of metal concentration measured in the bulk and on the surface. Therefore, it reflected the uncertainty of experimental measurements. The analysis of equivalence test is usually performed in two ways. We can construct a 100(1-2α)% confidence interval for the difference between the two mean values and compares it with [-θ, θ]. If the confidence interval is completely contained within the interval [-θ, θ], the mean values of the two data sets are equivalent. Alternatively, we can construct two one-side t-tests and take the greater p-value as p-value of the equivalence test.

$H_{0,a}: \Delta M_{OER} - \Delta M_{OER} \geq \theta$ (Supplementary Equation 7)

$H_{1,a}: \Delta M_{OER} - \Delta M_{OER} < \theta$ (Supplementary Equation 8)

and

$H_{0,b}: \Delta M_{OER} - \Delta M_{OER} \leq -\theta$ (Supplementary Equation 9)

$H_{1,b}: \Delta M_{OER} - \Delta M_{OER} > -\theta$ (Supplementary Equation 10)

These two methods are similar and in principle should give similar results. We carried out the t-test in our study.

Evaluation of Faradaic efficiency (FE) and solar to fuel (STF) efficiency

To calculate the FE, the composition of gas from the electrochemical cell was measured by gas chromatography. Knowing the concentration (*c*) of CO and H_2 and the gas flow rate (*r*), the FE is calculated as the ratio of the partial current corresponding to the generation of product to the total current (i_{total})

$FE_{CO} = {}^{r n_{CO} c_{CO} F}/_{I}$ (Supplementary Equation 11)

$FE_{H_2} = {}^{r n_{H_2} c_{H_2} F}/_{I}$ (Supplementary Equation 12)

where F is the Faraday constant, I is the total current, n_{CO} and n_{H_2} is the number of exchanged electrons to produce CO and H_2, respectively.

The STF efficiency is defined as the ratio of energy to burn the produced chemical to input solar energy. For CO and H_2, it is defined by

$$STF_{CO} = {V_{CO} I FE_{CO}}/{P_{solar} A} \quad \text{(Supplementary Equation 13)}$$

$$STF_{H_2} = {V_{H_2} I FE_{H_2}}/{P_{solar} A} \quad \text{(Supplementary Equation 14)}$$

$$STF = STF_{CO} + STF_{H_2} \quad \text{(Supplementary Equation 15)}$$

where P_{solar} is the incident solar power on the solar cell (100 $mW \cdot cm^{-2}$), A is the irradiated area of the solar cell. V_{CO} and V_{H_2} is the equilibrium potential for the reaction

$$CO_2 \rightarrow CO + 0.5O_2 \quad \text{(Supplementary Equation 16)}$$

$$H_2O \rightarrow H_2 + 0.5O_2, \quad \text{(Supplementary Equation 17)}$$

respectively. The values used in the calculations are: V_{CO} = 1.34 V and V_{H_2}=1.23 V.

Supplementary Note 1

The efficiency of solar-driven CO_2 reduction is determined by intersecting the J-V characteristics of the photovoltaic and electrolysis cell.[13] For the electrolysis cell, the current at the anode and cathode is a function of applied potential

$j_{CO2} = \boldsymbol{J^{CO2}}(\boldsymbol{V_{CO2}})$ (Supplementary Equation 18)

$j_{OER} = \boldsymbol{J^{OER}}(\boldsymbol{V_{OER}})$ (Supplementary Equation 19)

Here $\boldsymbol{J^{CO2}}$ and $\boldsymbol{J^{OER}}$ describes the potential dependent current density of CO_2 anode and SGC cathode, respectively. They are obtained either using the LSV result or the Tafel relation, the latter of which is

$\boldsymbol{J_{OER}} = j_{OER}^{0}\exp[\alpha(V_{OER} - V_{OER}^{0})]$ (Supplementary Equation 20)

The current densities of the photovoltaic, CO_2R-anode and OER-cathode satisfy

$j_{SC} \cdot A_{SC} = j_{CO2} \cdot A_{CO2} = j_{OER} \cdot A_{OER} = I$ (Supplementary Equation 21)

where A_{SC}, A_{CO2}, A_{OER} is the area of solar cell under irradiation, CO_2 electrode and OER electrode, respectively. A simplification is that the three components have the same area so that the system scalability and overall system cost are optimized. Under this simplification it requires

$j_{SC} = j_{CO2} = j_{OER}$ (Supplementary Equation 22)

Supplementary Equation 12, 14 and 16 give

$\boldsymbol{J_{CO2}}(V_{CO2} - V_{CO2}^{0}) = j_{OER}^{0}\exp[\alpha(V_{OER} - V_{OER}^{0})]$ (Supplementary Equation 23)

$V_{CO2} + V_{OER} = V$ (Supplementary Equation 24)

Solving Eq. S23 and S24 gives the J-V characteristic of the electrolysis cell. In our calculation, we used the data from Figure 3a for $\boldsymbol{J_{sc}}$, the reported LSC results for two CO_2 reduction anodes, CuAg nanocoral for the generation of oxygenate/hydrocarbon,[9] and sulfur modified tin for the generation of formate,[11] and the Tafel data in Figure 2b to obtain $\boldsymbol{J_{OER}}$. We note that we have

made several important assumptions in the estimation. First, we assume that the integration of CO_2R half-cell, OER half-cell and photovoltaic does not generate appreciable effect on the performance of any of these individual parts. Second, Supplementary Equation 24 neglects the voltage loss across the electrolysis cell so that the output photovoltage is the same as the input voltage to drive electrochemical reactions. The voltage loss is estimated to be ~0.1 V, which is significantly lower than the BPM-related voltage loss of 0.2-0.5 V. Third, we assume the same area of three components in the estimation.

Supplementary References:

1 Han, B. *et al.* Activity and stability trends of perovskite oxides for oxygen evolution catalysis at neutral pH. *Phys.Chem.Chem.Phys.* **17**, 22576-22580 (2015).

2 Man, I. C. *et al.* Universality in oxygen evolution electrocatalysis on oxide surfaces. *ChemCatChem* **3**, 11591--11165 (2011).

3 Yagi, S. *et al.* Covalency-reinforced oxygen evolution reaction catalyst. *Nat. Commun.* **6**, 8249 (2015).

4 McDonald, K. J. *et al.* Hydrothermal synthesis, structure refinement, and electrochemical characterization of Li_2CoGeO_4 as an oxygen evolution catalyst *J. Mater. Chem. A* **2**, 18428-18434 (2014).

5 Suntivich, J., May, K. J., Gasteiger, H. A., Goodenough, J. B. & Shao-Horn, Y. A perovskite oxide optimized for oxygen evolution catalysis from molecular orbital principles. *Science* **334**, 1383-1385 (2011).

6 Persson, K. A., Waldwick, B., Lazic, P. & Ceder, G. Prediction of solid-aqueous equilibria: Scheme to combine first-principles calculations of solids with experimental aqueous states. *Phys. Rev. B* **85**, 235438 (2012).

7 Jain, A. *et al.* The Materials Project: A materials genome approach to accelerating materials innovation. *APL Mater.* **1**, 011002 (2013).

8 Singh, A. K. *et al.* Electrochemical stability of metastable materials. *Chem. Mater.* **29**, 10159–10167 (2017).

9 Gurudayal *et al.* Efficient solar-driven electrochemical CO_2 reduction to hydrocarbons and oxygenates. *Energy Environ. Sci.* **10**, 2222-2230 (2017).

10 Zhou, X. *et al.* Solar-driven reduction of 1 atm of CO_2 to formate at 10% energy-conversion efficiency by use of a TiO_2-protected III–V tandem photoanode in conjunction with a bipolar membrane and a Pd/C Cathode. *ACS Energy Lett.* **1**, 764-770 (2016).

11 Zheng, X. *et al.* Sulfur-modulated tin sites enable highly selective Electrochemical reduction of CO_2 to formate. *Joule* **1**, 794-805 (2017).

12 Limentani, G. B., Ringo, M. C., Ye, F., Bergquist, M. L. & McSorley, E. O. Beyond the t-test: statistical equivalence testing. *Anal. Chem.* **77**, 221 A-226 A (2005).

13 Winkler, M. T., Cox, C. R., Nocera, D. G. & Buonassisi, T. Modeling integrated photovoltaic–electrochemical devices using steady-state equivalent circuits. *Proc. Natl. Acad. Sci.* **110**, E1076-E1082 (2013).
