# Supplementary material for: A high-performance oxygen evolution catalyst in neutral-pH for sunlight-driven CO2 reduction
Source: Nat Commun. 2019 Sep 9;10:4081. doi: 10.1038/s41467-019-12009-8 (PMC6733866; doi:10.1038/s41467-019-12009-8)
Supplement: Supplementary file 2 — Peer review [file 41467_2019_12009_MOESM2_ESM.pdf]

Reviewers' comments:

Reviewer #1 (Remarks to the Author):

I am satisfied by the responses and the additional characterizations provided by the authors. I think the manuscript now meets the standards of Nature Communications. The study is novel and the performances are remarkable. Acceptance for publication in Nature Communication is therefore recommended.

Damien Voiry.

Reviewer #2 (Remarks to the Author):

For characterizing the oxygen evolution reaction activity, the authors used glassy carbon disk electrode as substrate and IR-drop correction in this work, which is different from the vast literature that the samples are on carbon cloth/paper, nickel foam or other metal foil substrates. Thus, it is difficult to assess their performance. In addition, the authors used BET surface area rather than the commonly used ECSA or geometric area to calculate the current density, which further increases the difficulty for assessing the quality of this work. Furthermore, the common convention to define the overpotential is to use the current density of 10 mA/cm2, which is again different here. Since the authors have prepared the Sr2GaCoO5 anode by drop-casting a catalyst ink onto a carbon paper for the test of CO2 reduction, I suggest the authors tested their catalysts thoroughly on carbon paper substrate, and use geometric surface area and ECSA as a standard to calculate the current density. In this case, they could directly compare their catalysts with literature and provide a solid justification of the claimed excellent performance of their catalysts. In addition, they could test the stability of their catalysts for a very long time and at high current densities in this case, which is important for practical application. In the reviewer’s opinion, for earth-abundant catalysts, the absolute performance as an electrode is more meaningful than the mass activity or intrinsic activity as the cost does not change much as the loading increase. Based on the above, I suggest reconsideration after major revision. Please show the changes of texts and figures in the response to reviewer comments and mark the changes also in the manuscript and SI information for re-submission.

Reviewer #3 (Remarks to the Author):

I reviewed the manuscript NCOMMS-19-08231-T that was originally submitted to Nature Catalysis. I found that the quality of the manuscript has been improved largely. Several issues pointed out by the reviewers (including me) have been adequately addressed. Meanwhile, some issues are left unsolved even in the current version. I thus suggest the authors to make additional revisions. With appropriate revisions, I will reconsider this paper to be accepted for publication in Nature Communications.

(1)
The authors have concluded that the novel OER catalyst, Sr2GaCoO5 (SGC) exhibits chemical stability against possible OER-induced structural and compositional changes in neutral solutions. They have given XPS results (additional data in the revised manuscript) indicating that the surface composition of the SGC catalyst practically remains unchanged even after 100 CV cycles. In fact, the Ga 2p and Co 2p spectra of the fresh (as-prepared) and Ar-sputtered surfaces are essentially the same with each other (Figs. 3a and 3b). I am surprised to see the XPS data, because no signature of surface adsorption is evident for the soaked and 100 CV samples. The authors should present wide scan XPS data to validate that the spectra indeed come from very fresh surfaces, otherwise readers might guess that any pre-treatments (short-time Ar etching, etc.) were conducted prior to the XPS analyses.

(2)

The HRTEM images in Fig. 3e are conclusive evidences to rule out possible surface reconstruction in the SGC catalyst. Since this result is crucial to draw the main claim of this paper, I feel that a single image is not sufficient. I thus suggest the authors to present additional HRTEM images of different grains to demonstrate that the absence of an amorphous surface is a typical feature.

(3)
Regarding structural refinement of SGC, now I understand the authors' consideration. To support their claim, I suggest the authors to show simulation XRD patterns for the following structural models: one with perfect site preference of Co/Ga, the other with random distribution of Co/Ga. Also, an error should be given for each crystallographic parameter in Supplementary Table S2.

(4)
Additional comments:

Line 43. "oxygen evolution (OER)" should be corrected as "oxygen evolution reaction (OER)".

The numbering format of the Supplementary Information (SI) has not been corrected completely. Please be sure and modify the numbering format as "Fig. S1", "Fig. S2", etc. both in the main text and the SI.

Please give experimental details of XPS measurements: X-ray source, operation condition of the X-ray gun, type of conductive tape, operation condition of the Ar etching, etc.

Please correct some typos which have still left in the revised manuscript.
Line 99, "bounded" (bound)
Line 222, "magnetified" (magnified)
Line 245, "In in" (In)
Line 343, "grounded" (ground)

We deeply appreciate the reviewer for his careful reading and comments about our manuscript. All their comments have been seriously considered and the updated manuscript gives a clearer and better expression of our work.

Our major revisions include:

1. Measured the performance of SGC at 10 mA/cm^2 for 72 hours using carbon paper electrode (Reviewer 2)
2. Discussed the XPS results of Sr, C and O to show the influence of water adsorption after soaking or CV cycles (Reviewer 3)
3. Provided extra HRTEM images for SGC and SAC (Reviewer 3)
4. Confirmed the crystalline structure model from Rietveld refinement result (Reviewer 3)

Below we discuss each comment in details.

Reviewer #2:

For characterizing the oxygen evolution reaction activity, the authors used glassy carbon disk electrode as substrate and IR-drop correction in this work, which is different from the vast literature that the samples are on carbon cloth/paper, nickel foam or other metal foil substrates. Thus, it is difficult to assess their performance. In addition, the authors used BET surface area rather than the commonly used ECSA or geometric area to calculate the current density, which further increases the difficulty for assessing the quality of this work. Furthermore, the common convention to define the overpotential is to use the current density of 10 mA/cm2, which is again different here. Since the authors have prepared the Sr2GaCoO5 anode by drop-casting a catalyst ink onto a carbon paper for the test of CO2 reduction, I suggest the authors tested their catalysts thoroughly on carbon paper substrate, and use geometric surface area and ECSA as a standard to calculate the current density.

In this case, they could directly compare their catalysts with literature and provide a solid justification of the claimed excellent performance of their catalysts. In addition, they could test the stability of their catalysts for a very long time and at high current densities in this case, which is important for practical application. In the reviewer's opinion, for earth-abundant catalysts, the absolute performance as an electrode is more meaningful than the mass activity or intrinsic activity as the cost does not change much as the loading increase. Based on the above, I suggest reconsideration after major revision. Please show the changes of texts and figures in the response to reviewer comments and mark the changes also in the manuscript and SI information for re-submission.

Response: In the OER part of our paper, we used the glassy carbon rotating disk electrode to measure the oxygen evolution current and normalized it with the BET surface area to obtain the current density as a function of applied potential. For the purpose of evaluating the intrinsic activity, other resistances such as mass transport must be minimized. Thus, the GC-RDE setup is the most suitable as it avoids the diffusion of oxygen bubbles through porous electrode structures. In fact, the references that we compared the performance to all used the same GC-RDE setup in their experiments. Thus, we believe the glass carbon RDE provided the most reasonable comparison of the intrinsic activity with these literatures reports.

In order to address the Reviewer's comment, we measured the overpotential of carbon paper electrode at the geometric current density of 10 mA·cm$^{-2}{}_{geo}$. To keep consistent with the CO2 reduction experiment, we used the same loading of 1 mg·cm$^{-2}{}_{geo}$. The measured overpotential was

0.377 V and changed by less than 1 $mV\cdot hr^{-1}$ in 72 hours. Increasing the loading decreased the overpotential and for the loading of 3 $mg\cdot cm^{-2}{}_{geo}$ the overpotential was 0.348 V. We did not proceed to further increase the loading as it should require substantial engineering for minimizing the contact resistance and mass transfer resistance while keeping the integrity of electrode during operation. Nonetheless, the measured overpotential suggested the activity of SGC was still remarkable using carbon paper electrode.
We added the following paragraph to discuss these results. Following the reviewer's suggestion, the major changes are also highlighted in the manuscript.

While the GC-RDE setup allowed us to firmly establish the intrinsic activity and stability of SGC for oxygen evolution in neutral solution, we further evaluated the potential of using SGC towards practical applications. To do this we dropped casted the catalyst ink on a carbon paper electrode and measured the overpotential at the current density of 10 $mA\cdot cm^{-2}{}_{geo}$ in a three-electrode setup. For the catalyst loading of 1 $mg\cdot cm^{-2}{}_{geo}$, the measured overpotential was 0.377 V and barely changed in the testing of 72 hours (Supplementary Fig. 18). Due to the mass transfer resistance in the porous electrode, this value was about 0.05 V higher than that estimated from the GC-RDE measurements. Increasing the loading effectively decreased the overpotential required for the same current density. For the loading of 3 $mg\cdot cm^{-2}{}_{geo}$,[36] the overpotential was reduced to 0.348 V (Supplementary Fig. 17), only 0.018 V higher than the NiCoFeP oxyhydroxide catalyst. While engineering the loading and electrode structure to optimize the performance will be left to future work, these results clearly demonstrated the remarkable performance of SGC in conditions for practical applications.

Reviewer #3:
I reviewed the manuscript NCOMMS-19-08231-T that was originally submitted to Nature Catalysis. I found that the quality of the manuscript has been improved largely. Several issues pointed out by the reviewers (including me) have been adequately addressed. Meanwhile, some issues are left unsolved even in the current version. I thus suggest the authors to make additional revisions. With appropriate revisions, I will reconsider this paper to be accepted for publication in Nature Communications.
(1) The authors have concluded that the novel OER catalyst, Sr2GaCoO5 (SGC) exhibits chemical stability against possible OER-induced structural and compositional changes in neutral solutions. They have given XPS results (additional data in the revised manuscript) indicating that the surface composition of the SGC catalyst practically remains unchanged even after 100 CV cycles. In fact, the Ga 2p and Co 2p spectra of the fresh (as-prepared) and Ar-sputtered surfaces are essentially the same with each other (Figs. 3a and 3b). I am surprised to see the XPS data, because no signature of surface adsorption is evident for the soaked and 100 CV samples. The authors should present wide scan XPS data to validate that the spectra indeed come from very fresh surfaces, otherwise readers might guess that any pre-treatments (short-time Ar etching, etc.) were conducted prior to the XPS analyses.
Response: We appreciate the suggestion from the Reviewer. The XPS for the fresh surface was collected as prepared without any Ar etching. Indeed, the metal signals (Ga, Co and Sr) did not show any effect for the adsorption. The adsorption was more evident on the oxygen and carbon signals (supplementary Fig. 12). In the carbon spectra, the pristine material displayed weak signal due to the adsorption of atmospheric CO2, while the soaked and CV cycled samples showed much stronger signals from catalyst ink. In the oxygen spectra, we see the clear peak at 532.2 eV due to

the adsorbed OH group. These results confirmed that the contact with aqueous electrolyte mainly induced changes on C and O spectra, but not for metal signals.

We now added the Sr data to Figure 3 to further demonstrate the little effect of water adsorption and electrochemical operation on metal species. The carbon and oxygen data is added as Supplementary Fig. 12.

(2) *The HRTEM images in Fig. 3e are conclusive evidences to rule out possible surface reconstruction in the SGC catalyst. Since this result is crucial to draw the main claim of this paper, I feel that a single image is not sufficient. I thus suggest the authors to present additional HRTEM images of different grains to demonstrate that the absence of an amorphous surface is a typical feature.*
Response: We agree with the Reviewer that HRTEM is one of the crucial evidence in our study. Thus, we have added new HRTEM images for SGC and SAC after 100 CVs, each taken for two different grains (Supplementary Fig. 14). In all three HRTEM images for SGC (including the original one from Fig. 3), we see the crystalline fringe extended all the way to the surface boundaries, while for SAC an amorphous surface layer is clear.

(3) *Regarding structural refinement of SGC, now I understand the authors' consideration. To support their claim, I suggest the authors to show simulation XRD patterns for the following structural models: one with perfect site preference of Co/Ga, the other with random distribution of Co/Ga. Also, an error should be given for each crystallographic parameter in Supplementary Table S2.*
Response: We have followed the suggestion of Reviewer 3 to simulate the XRD patterns for SGC with ordered Co/Ga occupancy and random distribution of Co/Ga. The result is shown in the figure below. The major difference is the enhanced intensities at around 11.5°, 23°, 39.5°-41.5°, and 52° for random Co/Ga distribution. These correspond to the diffraction from (020), (130), (240), (042), (161), (181) planes. Because the Co/Ga plane is parallel to (010), we expect the distribution of Co/Ga affects the diffraction from planes in parallel with (010), which explains the observed difference. Compared to the experimental XRD pattern (Figure 1a), it is apparent that the simulated result from ordered Co/Ga agrees better, which supports the nearly ordered occupancy of Co and Ga.
The error for the refined crystallographic parameters is provided in Supplementary Table S2.

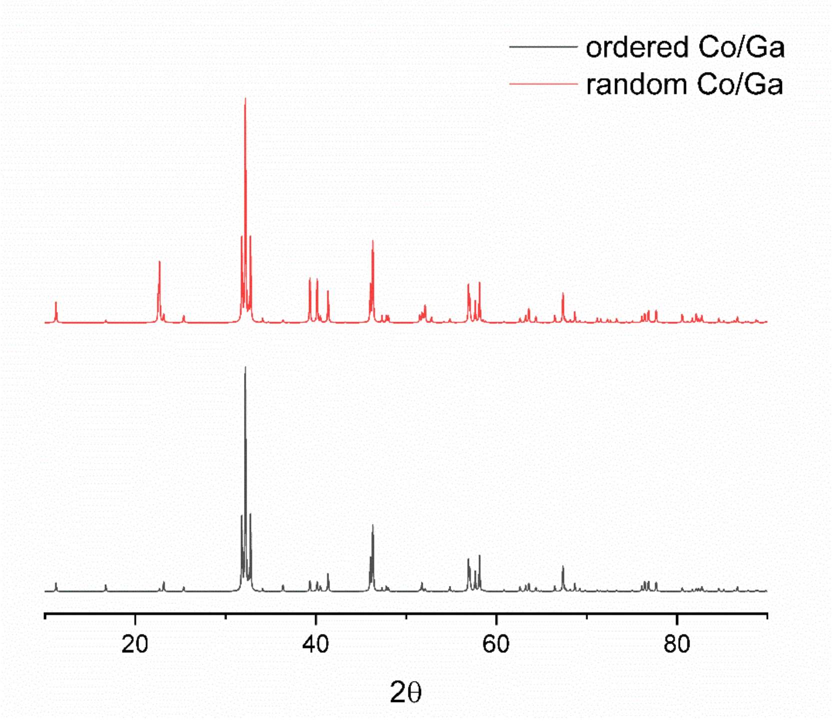

(4) Additional comments:
Line 43. "oxygen evolution (OER)" should be corrected as "oxygen evolution reaction (OER)".
Response: We have corrected this typo.
The numbering format of the Supplementary Information (SI) has not been corrected completely. Please be sure and modify the numbering format as "Fig. S1", "Fig. S2", etc. both in the main text and the SI.
Response: We have corrected the formatting of figure captions in the supplementary information as well as in the manuscript when it is mentioned. For the captions it seems that the format should be “Supplementary Fig. 1”, which we used in the revision.

Please give experimental details of XPS measurements: X-ray source, operation condition of the X-ray gun, type of conductive tape, operation condition of the Ar etching, etc.
Response: We have added the experimental information for XPS measurements.

X-ray photoelectron spectroscopy (XPS) spectra were collected with a PHI 5000 VersaProbe II X-ray photoelectron spectrometer using an Al Kα source. The X-ray parameter conditions were 15 kV, 25 W, pass energy of 23.5 eV and at a resolution of 0.2 eV/step. The sample was mounted on double-sided carbon tape and tilted at 45 degrees. An alternating Ar+ ion source was used for sputtering at 1 kV.

Please correct some typos which have still left in the revised manuscript.
Line 99, "bounded" (bound)
Line 222, "magnetified" (magnified)
Line 245, "In in" (In)
Line 343, "grounded" (ground)

Response: We have corrected these typos.

Reviewers' comments:

Reviewer #2 (Remarks to the Author):

The authors have addressed most of my comments, and the quality of the manuscript has been greatly improved. It can be accepted after further minor revision. The authors should provide the detailed LSV curves of the catalysts loaded on the carbon paper electrode. In addition, they should provide the stability data at 100 mA cm-2.

Reviewer #3 (Remarks to the Author):

I reviewed the manuscript NCOMMS-19-08231A, the revised version of NCOMMS-19-08231-T by Zhou et al. I found that the quality of the manuscript has been further improved. I am mostly satisfied with the revisions relating to the XPS result and editing errors. Also, it seems, in my opinion, that the authors' responses to the comments of Reviewer #2 are likely reasonable: the stability test employing a carbon paper electrode is particularly impressive.

Nevertheless, I feel that the manuscript still contains critical issues which are related to the crystallographic characterization and microstructural observations. For details, please see the following comments. I conclude that additional revisions must be made before this paper is accepted for publication in Nature Communications.

(1) Structural refinement of SGC
In response to my previous comment, the authors present simulated XRD patterns for the two structural models: one with perfect site preference of Co/Ga, the other with random distribution of Co/Ga. While the two patterns are indeed distinguishable to each other, I am surprised to see that the difference is so clear. To confirm the validity of these simulated patterns, I tentatively performed diffraction simulations by myself. My own result gives a rather different aspect: the two patters are very similar to each other, and this result rather agrees with my anticipation.

I suggest the authors to carefully check their diffraction simulations. The re-calculation should be done (if possible) employing other refinement programs. It should be emphasized that the crystallographic feature of SGC is important, because the authors' mechanistic considerations rely on the electronic/crystal structures.

(2) HRTEM observations
In response to my previous comment, the authors give additional HRTEM images for SGC after 100 CVs (Supplementary Figs. 14a and 14b). While the two images look similar in terms of magnification, the scale bars put in these images are different in size. At a glance, the scale bar in Fig. 14b is 20% larger than the one in Fig. 14a. Please check carefully whether the images have been processed adequately. The authors should pay close attention to their data management, because such an error is unfavorably impressed.

Reviewer #2 (Remarks to the Author):

The authors have addressed most of my comments, and the quality of the manuscript has been greatly improved. It can be accepted after further minor revision. The authors should provide the detailed LSV curves of the catalysts loaded on the carbon paper electrode. In addition, they should provide the stability data at 100 mA cm-2.

Response: We have measured the LSV and the high current density performance and added the results in Supplementary Fig. 18. As we expected, the stable performance persisted even at high current densities. However, we make cautious to the readers that at high current densities the iR correction became significant and the carbon electrode may be vulnerable for oxidation at high voltages.

Reviewer #3 (Remarks to the Author):

(1) Structural refinement of SGC

In response to my previous comment, the authors present simulated XRD patterns for the two structural models: one with perfect site preference of Co/Ga, the other with random distribution of Co/Ga. While the two patterns are indeed distinguishable to each other, I am surprised to see that the difference is so clear. To confirm the validity of these simulated patterns, I tentatively performed diffraction simulations by myself. My own result gives a rather different aspect: the two patters are very similar to each other, and this result rather agrees with my anticipation.

I suggest the authors to carefully check their diffraction simulations. The re-calculation should be done (if possible) employing other refinement programs. It should be emphasized that the crystallographic feature of SGC is important, because the authors' mechanistic considerations rely on the electronic/crystal structures.

Response: We appreciate the reviewer's careful examination. The program to simulate the XRD was Mercury downloaded from the Cambridge Crystallographic center (https://www.ccdc.cam.ac.uk/solutions/csd-system/components/mercury/). The result given by the reviewer made us cautious about the simulation and eventually it seemed that the version we used cannot treat the partial occupancy well. We then used VESTA and got the same results as the reviewer, as shown in the figure below.

We performed the simulation for four structures: ordered Co/Ga as we reported in the paper, random Co/Ga, and ordered Ga/Co with site switched. These three models had the same structural parameter and only differed on the occupancy at Co and Ga sites. The fourth model is the structure refined with random Co/Ga occupancy. The simulated XRD spectra are shown in the Figure below. We do observe the difference at several positions, as marked by the arrows. In general, switching the positions for Ga and Co increases the intensity at 11.22° and 41.35° while decreasing at 25.37°. To examine which one agreed with the experiment better, the intensities are compared with the experimental data in the Table. We clearly see that the ordered Co/Ga indeed matched the best with the experiments for these peaks. Although the refined random occupancy also gives a reasonable agreement, we note that after considering all other peaks the R_{wp} and R_p was increased by 2% and 1.7% compared to ordered Co/Ga, respectively.

We therefore conclude that the ordered Co/Ga as we reported in the paper is the best model for the experimental XRD data.

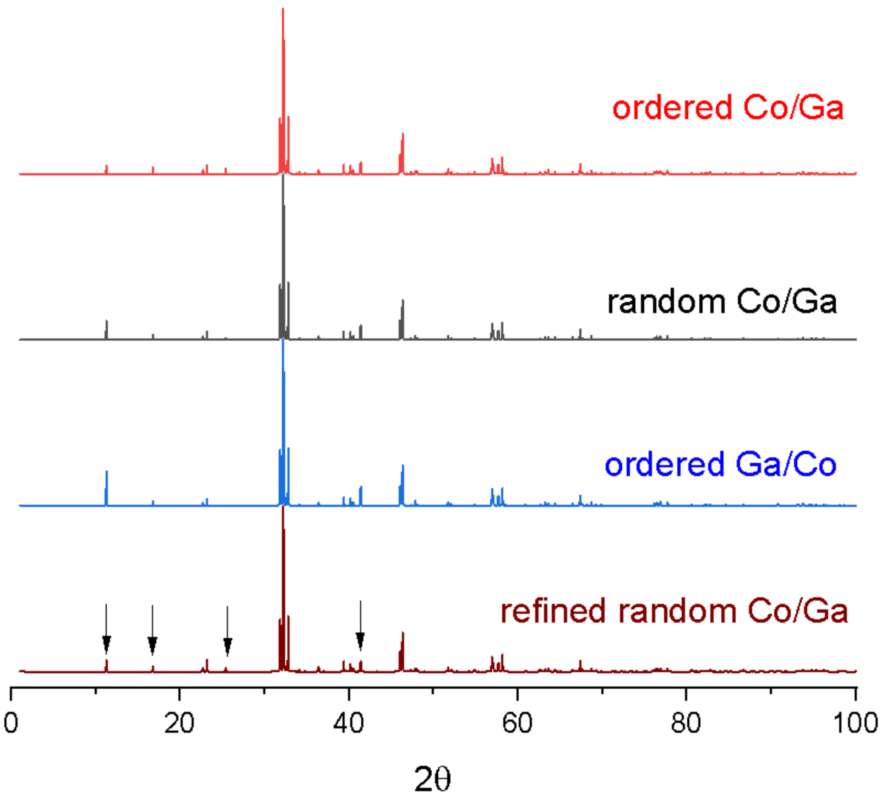

Table 1. Simulated XRD intensities and experimental data. The experimental data is normalized to the highest intensity at 31.18°.

angle	11.22° (020) plane	16.73° (110) plane	25.37° (121) plane	41.35° (161) plane
Ordered Co/Ga	5.32	3.87	3.5	6.96
Random Co/Ga	11.33	3.08	1.00	9.04
Ordered Ga/Co	20.89	2.41	0.27	41.37
angle	11.22°	16.73°	25.37°	41.36°
Random refined	6.97	3.25	2.09	6.35
angle	11.20°	16.68°	25.35°	41.37°
experiment	7.02	3.22	3.44	9.72

Table 2. Refined crystallographic parameters for Sr_2GaCoO_5 from Rietveld refinement assuming random Co/Ga occupancy.

atom	x	y	z	Occupancy	U_{iso}
Sr	0.0167(2)	0.1134(0)	0.5	1.0000	8
Co1	0	0	0	0.784(5)	4
Ga1	0	0	0	0.216(5)	4
Co2	-0.0679(3)	0.25	-0.0352(6)	0.108(5)	8
Ga2	-0.0679(3)	0.25	-0.0352 (6)	0.392(4)	8
O1	0.25	-0.0045(4)	0.25	1.0000	8
O2	0.0349(9)	0.1412(2)	0	1.0000	8
O3	0.8787(15)	0.25	0.6244(15)	0.5000	8

R_{wp} = 11.74%, R_p = 8.96%, χ^2 = 1.615, a = 5.6225(1) Å, b = 15.7577(3) Å, c = 5.4620(1) Å, V = 483.92(3) Å^3, ρ_{calc} = 5.269 g/cm^3. The space group is Icmm.

(2) HRTEM observations

In response to my previous comment, the authors give additional HRTEM images for SGC after 100 CVs (Supplementary Figs. 14a and 14b). While the two images look similar in terms of magnification, the scale bars put in these images are different in size. At a glance, the scale bar in Fig. 14b is 20% larger than the one in Fig. 14a. Please check carefully whether the images have been processed adequately. The authors should pay close attention to their data management, because such an error is unfavorably impressed.

Response: Again, the careful examination of the reviewer is much appreciated. In fact, the images in Figure S14 is used as it is without further processing (except that we grouped four images into one Figure). The magnifications are indeed different, which is also seen from the size information in the .dm3 files.

In the slides, we provided four images, S14a and the same particle with lower magnification (14a'); S14b and S14b', which was taken slightly on the right side of S14b and at the same magnification as Fig. S14a. We then counted the number of spots along two different directions. The results are very consistent, and the largest difference is 1 for every 16 spots, which we consider to be reasonable. For 14a and 14b' at the same magnification, the numbers of spots are the same for both directions.
However, the counting along the horizonal direction should be taken with care, because it contains alternate bright and dark spots, and some dark spots may look quite bright. This may cause the large difference (16 vs 20) as counted by the reviewer.

When analyzing these figures, the coincidence that S14a and S14b had the same crystalline orientation as shown from the SAED made us realize that the evidence may not be sufficient to judge whether they represented two different particles. We measured the size of S14a' and found that that image was not big enough to include the kinked part in S14b' for a conclusion. Therefore, to avoid confusion, we made the following changes to Figure 3 and Figure S14:
(1). We replace the cycled sample in Figure 3c with Figure S14a, because the SAED for S14a provided additional evidence for the crystallinity of the cycled sample.
(2). We removed S14b from Figure S14, and only used the original Figure 3c as the new Figure S14a. The caption is also revised accordingly.
(3). We provided two new HRTEM figures for the cycled SGC as the new Figure S14b and S14c.

(4). We provide a powerpoint file with S14a, S14a’ S14b, S14b’ for the reviewing purpose. All the images in this file are in their original formats without any edition.

Reviewers' comments:

Reviewer #2 (Remarks to the Author):

The 2 h high current density measurement is not long enough. The authors should perform longer measurement. In addition, the iR-correction of the LSV curve is absurd. The current can not be the same in different applied potentials. The purpose of such LSV and stability measurement is to see the absolute performance of the catalyst in real electrode conditions. Thus, iR-correction is not needed. The manuscript can be accepted after addressing the above comments.

Reviewer #3 (Remarks to the Author):

I reviewed the manuscript NCOMMS-19-08231B, the 2nd revised version of NCOMMS-19-08231-T by Zhou et al. I found that the authors have addressed most of the comments by the reviewers (including me). I am mostly satisfied with the authors' explanations for the TEM images, and their response regarding the XRD issue seems to be reasonable, although I am a little worried why the goodness of fit (R factor) was significantly deteriorated (Rwp = 9.73% to 11.74%) when removing a restriction for the Co/Ga occupancy: this seems to be an opposite consequence. When a clear aspect is given by the authors, I will recommend this paper to be accepted for publication in Nature Communications.

Reviewer #2 (Remarks to the Author):

The 2 h high current density measurement is not long enough. The authors should perform longer measurement. In addition, the iR-correction of the LSV curve is absurd. The current can not be the same in different applied potentials. The purpose of such LSV and stability measurement is to see the absolute performance of the catalyst in real electrode conditions. Thus, iR-correction is not needed. The manuscript can be accepted after addressing the above comments.

Response: As the Reviewer 2 suggested, we measured the performance at 100 mA/cm2 for 32 hours. The voltage increased by less than 0.015 V during this period. We also agreed with the Reviewer 2 that for the carbon paper electrode the iR correction is not necessary. Thus, we added the discussion in the manuscript as "Even at a high current density of 100 mA·cm$^{-2}{}_{geo}$, the voltages remained highly stable with an increase of less than 0.015 V in the testing of 32 hours." and revised Supplementary Fig. 18

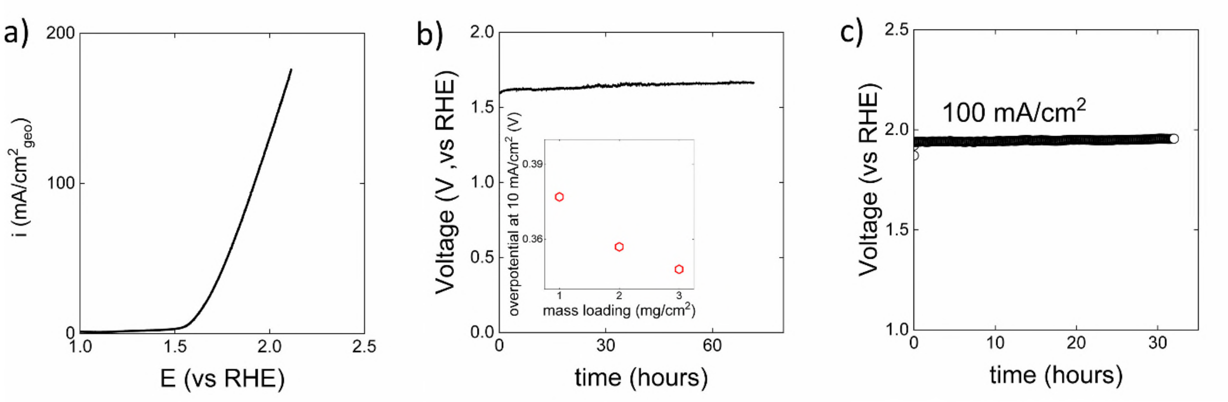

Supplementary Fig. 18. Performance of a carbon paper electrode loaded with Sr_2GaCoO_5 for oxygen evolution reaction in neutral pH. (a) Linear sweep voltammogram of the carbon paper electrode. The loading of catalyst was 1 mg·cm^{-2}. (b) Overpotential of the carbon paper electrode for 72 hours of operation at 10 mA·cm^{-2}. The overpotential was 0.377 V and increased by less than 1 mV per hour. The insertion showed the measured overpotential at the same geometric current density for different loadings. (c) Voltages of the carbon paper electrode at 100 mA. The loading of catalyst was 1 mg·cm^{-2}. At high current densities the effect of resistant increases. At 100 mA·cm^{-2}, the measured resistance caused 0.245 V loss of voltage for a 0.5 cm^2 electrode (R=4.9 Ω in LSV measurement and 4.8 Ω in galvanostatic measurements). Note at such high voltages the carbon electrode may be vulnerable for oxidation.

Reviewer #3 (Remarks to the Author):

I reviewed the manuscript NCOMMS-19-08231B, the 2nd revised version of NCOMMS-19-08231-T by Zhou et al. I found that the authors have addressed most of the comments by the reviewers (including me). I am mostly satisfied with the authors' explanations for the TEM images, and their response regarding the XRD issue seems to be reasonable, although I am a little worried why the goodness of fit (R factor) was significantly deteriorated (Rwp = 9.73% to 11.74%) when removing a restriction for the Co/Ga occupancy: this seems to be an opposite consequence. When a clear aspect is given by the authors, I will recommend this paper to be accepted for publication in Nature Communications.

Response: We completely agreed with the Reviewer 3 that mathematically the R_{wp}, a measurement of weighted mean square error of the fitting, should decrease if the degree of freedom in the fitting increases.

In our case it seems to correspond to relax the occupancy of Co and Ga site. However, we want to clarify that the relaxation should satisfy other constraints, such as the occupancy should be no less than 0 and no higher than 1, and the sum of Co and Ga at each site should be 1. Imposing these constraints allows us to fit the diffraction with a physically reasonable model. On the other side it may cause the model to converge at a local optimum with increased R_{wp}.
To explain the question from the Reviewer 3, we refined the structure again without imposing any constraints on the site occupancy. The results are shown in the Table below. In completely agreement with the Reviewer 3's expectation (also ours), the fitting converged with a slightly lower R_{wp} than that we reported in the paper (9.69% vs 9.73%). However, the target of refinement is to provide a physically meaningful model that explains not only the XRD but also other characterizations such as HAADF-STEM, instead of pursuing lower R_{wp}. Considering the Co1/Ga1 or Co2/Ga2 sites are no longer fully occupied in the new model, we conclude that the model in the manuscript is more physically reasonable although the R_{wp} is 0.04% higher.

Atom	x	y	z	Occupancy	Multiplicity	U_{iso}
Sr	0.0167(2)	0.1118(0)	0.5	1.0000	8	0.0024(3)
Co1	0	0	0	0.328(7)	4	0.0033(8)
Ga1	0	0	0	0.516(6)	4	0.0033(8)
Co2	-0.0711(3)	0.25	-0.0366(7)	0.356(4)	8	0.025(1)
Ga2	-0.0711(3)	0.25	-0.0366(7)	0.246(3)	8	0.025(1)
O1	0.25	-0.0073(3)	0.25	1.0000	8	0.0015(14)
O2	0.0351(8)	0.1367(2)	0	1.0000	8	0.0026(14)
O3	0.9133(14)	0.25	0.6291(14)	0.5000	8	0.016(3)

R_{wp} = 9.69%, R_p = 7.32%, χ^2 = 1.291, a = 5.6221(1) Å, b = 15.7579(3) Å, c = 5.4621(1) Å, V = 483.90(2) Å^3, ρ_{calc} = 5.310 g/cm^3

Reviewers' comments:

Reviewer #2 (Remarks to the Author):

The authors have addressed my comments, and the manuscript can be accepted for publication now.

Reviewer #3 (Remarks to the Author):

I reviewed the manuscript NCOMMS-19-08231C, the 3rd revised version of NCOMMS-19-08231-T by Zhou et al. Unfortunately, my concern regarding the structural refinement has remained unsettled. I thus suggest the authors to reconsider their logical pathway in the discussion on the OER mechanism of Sr_2GaCoO_5.

The refinement results, in which the Co/Ga ordered and disordered models have given similar scores (R_{wp} = 9.73% and 9.69%, respectively), imply that XRD studies do not provide definitive clues to the Co/Ga occupancies. Note that I pointed out this remark already in the 1st reviewing stage (NATCATAL-18111624). HAADF-STEM would hardly distinguish between cobalt and gallium because of their similar atomic numbers. Also, the larger magnetic moment of SGC than $LaCoO_3$ may be explained assuming the existence of HS Co^{3+} at the tetrahedral site. Thus, I believe that the authors cannot exclude the Co/Ga disordered model at this stage.

For further revisions, I suggest two choices. The authors should prove the validity of the Co/Gd ordered model by means of, for instance, XAFS and/or STEM-EDX. Otherwise, additional mechanistic studies need be conducted assuming Co/Ga disordered model to theoretically clarify how the Co/Ga mixing influences the electronic structure of SGC and thereby the energy diagram for oxygen evolution.

Reviewer #3 (Remarks to the Author):

I reviewed the manuscript NCOMMS-19-08231C, the 3rd revised version of NCOMMS-19-08231-T by Zhou et al. Unfortunately, my concern regarding the structural refinement has remained unsettled. I thus suggest the authors to reconsider their logical pathway in the discussion on the OER mechanism of Sr2GaCoO5.

The refinement results, in which the Co/Ga ordered and disordered models have given similar scores (Rwp = 9.73% and 9.69%, respectively), imply that XRD studies do not provide definitive clues to the Co/Ga occupancies. Note that I pointed out this remark already in the 1st reviewing stage (NATCATAL-18111624). HAADF-STEM would hardly distinguish between cobalt and gallium because of their similar atomic numbers. Also, the larger magnetic moment of SGC than LaCoO3 may be explained assuming the existence of HS Co3+ at the tetrahedral site. Thus, I believe that the authors cannot exclude the Co/Ga disordered model at this stage.

For further revisions, I suggest two choices. The authors should prove the validity of the Co/Gd ordered model by means of, for instance, XAFS and/or STEM-EDX. Otherwise, additional mechanistic studies need be conducted assuming Co/Ga disordered model to theoretically clarify how the Co/Ga mixing influences the electronic structure of SGC and thereby the energy diagram for oxygen evolution.

Response: As we had explained in the previous response, the reason that we ruled out the random Ga/Co model is due to the unphysical structure after refinement. In the Icmm Brownmillerite structure, small sized cations reside either in the 4a (0, 0, 0) octahedral or 4e (x, 0.25, 0) tetrahedral site. The number of available tetrahedral and octahedral site is 1:1. In our case the tetrahedral site is distorted so that the actual position is 8i (x,0.25, z). However, the displacement of z is small and we should not anticipate that two ions simultaneously occupies at the same (x, 0.25, z) and (x,0.25,-z). This gives a physical limitation that the occupancy of 8i should be strictly no larger than 0.5.

However, the refinement of the random Ga/Co model violates this physical restriction that the occupancy at Ga2/Co2 site is 0.356+0.246=0.602. This indicates 20% of tetrahedral sites are occupied by two cations with a distance of 0.36 Å. This is the reason that we ruled out this unphysical structure.

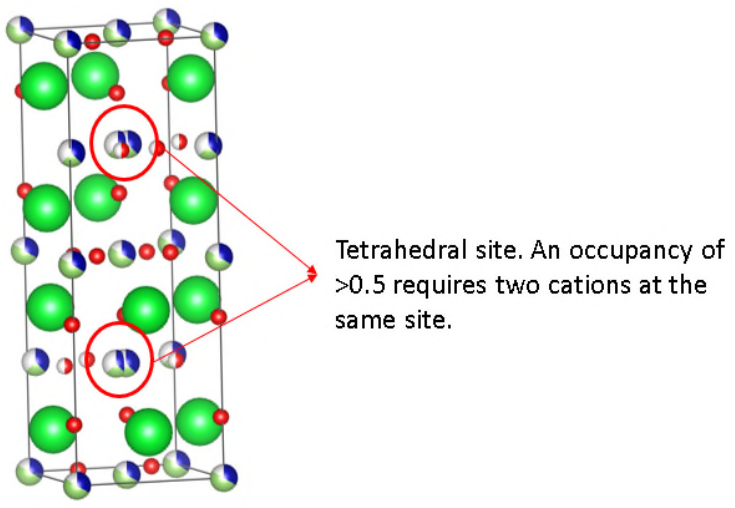

To briefly summarize all the discussions related to the structural model, we had considered three models in the refinement. The random Ga/Co model without restriction yielded the lowest Rwp = 9.69%. But it gave unphysical occupancy at the tetrahedral site and thus was ruled out. The random Ga/Co model with restriction on site occupancy yielded much higher Rwp = 11.74% and thus was ruled out. Only the ordered Ga/Co model provided the best physical structure as well as a Rwp only 0.04% higher than the lowest value. This is the reason we concluded the ordered Ga/Co occupancy in SGC.
